# Primary Health Care Systems and Their Contribution to Universal Health Coverage and Improved Health Status in Seven Countries: An Explanatory Mixed-Methods Review

**DOI:** 10.3390/ijerph21121601

**Published:** 2024-11-30

**Authors:** Anjana Rai, Resham B. Khatri, Yibeltal Assefa

**Affiliations:** 1 School of Public Health, University of Queensland, Herston, QLD 4006, Australia; r.khatri@uq.edu.au (R.B.K.); y.alemu@uq.edu.au (Y.A.); 2 School of Public Health and Social Work, Queensland University of Technology, Kelvin Grove, QLD 4059, Australia

**Keywords:** primary health care, health systems, service delivery, health workforce, health information system, health financing, medicines, leadership and governance, community participation, multisectoral actions

## Abstract

Background: Primary health care (PHC) systems and their successes and challenges vary between and within countries. We elucidate the role of PHC on health status and universal health coverage (UHC) by describing the achievements and challenges of PHC systems in seven countries representing the three economic levels: high-income (Belgium, Australia), middle-income (South Africa, Thailand), and low-income countries (Cambodia, Ethiopia, and Nepal). Methods: We adopted a mixed-methods approach and (a) extracted quantitative data on the key health and universal health coverage index of countries and (b) conducted a scoping review of the PHC systems in these countries. We used key terms related to the following eight domains: service delivery, health workforce, health information system, health financing, medicines, and leadership and governance (the WHO’s building blocks for national health systems) and community participation and multisectoral actions (other pillars of PHC) to identify the relevant literature and searched six databases: PubMed, Scopus, Embase, PsycINFO, CINAHL, and Cochrane Library. A total of 58 articles were identified and included in this review; data were charted and synthesised narratively. Results: There is variation in health services coverage and health status across the three economic levels. Countries expanded access to PHC services using strategies like telehealth and CHWs but faced challenges in sustainability, workforce retention, and service quality. Community engagement and multisectoral actions helped, though gaps in governance, resources, and essential medicines hindered progress towards UHC. Conclusions: By addressing the challenges and leveraging successful strategies, countries can move closer to achieving the goal of universal health coverage and improving health outcomes for all.

## 1. Introduction

Primary health care (PHC) is the first point of contact with the health system for people seeking health services. A comprehensive PHC focuses on disease prevention, health promotion, treatment, care, and rehabilitation. Providing accessible, comprehensive, and people-centred health services across the lifespan while addressing social, economic, and environmental determinants of health is fundamental to the successful implementation of the PHC approach [1]. A PHC-oriented health system promotes health equity, reduces health disparities, and produces better outcomes. The six key building blocks of health systems: service delivery, health workforce, health information system, health financing, medicines, and leadership and governance [2] are equally important to strengthen PHC towards meeting population health needs. PHC goes beyond conventional health concerns and seeks to improve health and reduce inequity through multisectoral actions and community participation by understanding and addressing the multiple social determinants of health and empowering communities [3].

PHC is central to the efforts to achieve the health targets of the Sustainable Development Goals 3 (SDG 3) and sub-goal 3.8 of universal health coverage (UHC) [4,5,6]. The Alma Ata Declaration 1978 and the Astana Declaration 2018 acknowledge the fundamental role of PHC in improving population health [3,7]. Even though several factors within and beyond the health sector are critical in shaping health and wellbeing, there is a consensus that the expansion and strengthening of PHC are associated with improved population health [3,4,5,8]. Universal health coverage includes financial risk protection, access to quality essential health-care services, and access to safe, effective, quality, and affordable essential medicines and vaccines for all [9]. Addressing the wider determinants of health, promoting equity and social justice, and empowering communities through PHC systems can advance the achievement of universal health coverage and the SDGs [10].

After the World Health Organisation (WHO) highlighted the importance of UHC in 2010, countries have been strengthening PHC systems to deliver maternal and child health and reach underserved populations [11]. PHC strengthening has contributed to increased coverage, additional health services, and access to and utilisation of health services [4,8,12]. Existing studies have further noted current initiatives in community engagement and multisectoral actions on PHC [5,13,14]. However, a comprehensive evidence synthesis incorporating other health system domains crucial to implementing PHC is needed. Furthermore, PHC systems and their successes and challenges vary between and within countries. Learning about successes and challenges in PHC implementation allows programme and policymakers to adapt strategies that work and address challenges for an efficient PHC design that ultimately contributes to UHC and health.

Although we are unable to directly relate quality of PHC to UHC and health status using the review methodology, there is a consensus that quality PHC systems improve people’s health and wellbeing [3], and PHC is central to achieving UHC [4]. Based on this premise, the aim of this explanatory mixed-methods review was to describe the UHC and health status of selected countries and scope the significance of their PHC systems.

## 2. Materials and Methods

### 2.1. Study Design

This study is a mixed-methods review using multiple designs. On the premise that PHC contributes to UHC and improved health status, first, we purposefully selected countries based on their performance on the UHC index and globally comparable health indicators. As a global review was beyond the scope, we selected seven countries: Belgium, Australia (high-income); South Africa, Thailand (upper-middle-income); Cambodia, Ethiopia, and Nepal (low and lower-middle-income) [15]. We extracted quantitative data on the key health indicators and UHC indices of these countries.

This was followed by a scoping review of the literature published in academic databases to explore the successes and challenges of PHC systems and their contribution to UHC and health [16]. We used the WHO six building blocks: service delivery, health workforce, health information system, health financing, medicines, and leadership and governance [2]; and added community participation and multisectoral actions from the Astana declaration, resulting in eight total domains [3]. This two-step method enabled us to explore how PHC systems are different between the countries performing differently in the UHC index and health indicators and to identify successes and challenges for programme and policy implications.

### 2.2. Identifying the Research Question

We were interested in how different countries’ PHC systems function, successes and challenges in PHC, and how they contribute to UHC and health.

### 2.3. Search Strategy

To identify relevant studies on PHC, we searched six databases: PubMed, Scopus, Embase, PsycINFO, CINAHL, and Cochrane Library. The search strategy involved a few stages:

1. We ran a search on Pubmed and reviewed the first 100 titles and abstracts to identify PHC-related keywords used.
2. We built the search terms in stages and included terms related to six health system blocks: service delivery, followed by health workforce, health information system, essential medicines and vaccination, health financing, and governance.
3. We added terms related to health systems, community engagement, multisectoral actions, and seven countries’ names.

Boolean operators (AND/OR) and truncations (“”, *) were used depending on the database. The search included all studies published in English from 1 January 2000 until 31 December 2023. The total hits from the database searches were 6537 articles. Supplementary Document S1 shows the search strategy utilised in this review. The articles from each database search were imported to endnote, and 2620 duplicate records were removed. The remaining records were screened for titles and abstracts by AR and RK. This was followed by full-text articles screening by AR.

### 2.4. Eligibility Criteria

**Figure 1:**
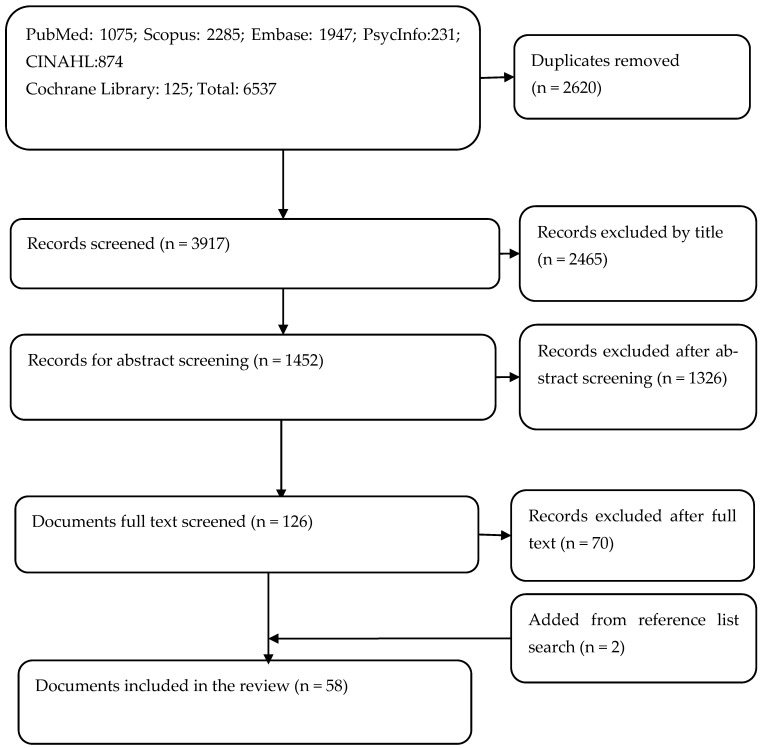
Selection of articles included in this review.

Our inclusion and exclusion criteria considered the population (health service users, care providers, and managers), concept (PHC), and contexts (seven countries). This review included all qualitative, quantitative, or mixed-methods studies and the grey literature with findings relevant to our objective. The consort flow chart in Figure 1 shows the study selection process utilised in this review.

### 2.5. Data Charting and Synthesis

We charted data on author, year, country, type of study, and main findings (Supplementary Table S1). The findings were then summarised by eight domains: service delivery, health workforce, health information system, health financing, medicines, leadership and governance, community participation, and multisectoral actions.

## 3. Results

A total of 3917 titles were screened, followed by 1452 abstracts. In total, 126 full-text articles were screened, removing 70 articles. Additionally, we searched the reference list of relevant articles and identified two articles, resulting in a total of 58 documents included in this review (Figure 1).

Table 1 presents the selected health indicators of the seven countries under review as per the World Bank classification [15]. It also shows the overall UHC index and specific UHC index for reproductive, maternal, and child health, infectious diseases, and non-communicable disease services in the seven countries under review. High-income countries such as Belgium and Australia have better health indicators and UHC coverage. Although upper-middle-income, South Africa’s health indicators and UHC index are closer to those of the low-income countries under review, particularly for life expectancy and child mortality rates. The UHC indices also resemble low-income settings. Thailand’s health indicators and UHC indices are relatively better than those of low-income countries and are closer to Belgium’s and Australia’s indices. Among low-income countries, health indicators and UHC indices are similar, but Cambodia has comparatively better health indicators and UHC than Ethiopia and Nepal. Table 2 provides the summary of findings on the successes and challenges of PHC systems.

### 3.1. Service Delivery

Countries adopted strategies to provide PHC services to remote and vulnerable populations. These strategies expanded service access in hard-to-reach populations and remote and regional areas, thereby increasing coverage and addressing inequality in service access, which is crucial for improving UHC. However, persistent challenges created gaps in the achievement of UHC. For example, in Australia, several initiatives were implemented: telehealth services [21,22], priority setting for the Indigenous population, enhancing cultural safety [19], recruitment of Aboriginal and/or Torres Strait Islander health workers (A&TSIHWs) [20], community participation [60], coordinated care [18], and addressing barriers related to distance [21]. These strategies effectively increased the coverage of PHC services in remote and rural areas and among Aboriginal and/or Torres Strait Islander people. However, challenges remained, particularly regarding the range and quality of the services provided. Rural and remote areas, compared to urban areas, had fewer GPs, PHC services were less affordable, and after-hours care was limited compared to major cities [18]. Even though the telehealth strategy extended access to remote and rural areas, it was resource-intensive and increased staff workload [22], raising concerns about sustainability. Additionally, such programmes may exclude populations not owning smartphones [22], widening the health inequality gap between certain sub-populations.

In Belgium, the piloting of CHWs successfully reached socio-economically vulnerable communities and facilitated access to PHC [23]. CHWs also played a crucial role in helping people navigate the digital technologies required to access health services [23], minimising the service access gap created by using digital technology in PHC services.

In South Africa, community-based programs such as CHWs and ward-based outreach teams were instrumental in increasing PHC coverage by providing health care services to underserved communities and facilitating the link between their communities and health care facilities [26,27,28]. Key factors contributing to the success of these programmes included comprehensiveness, coordination, the cultural competency and availability of the PHC team [24], and structures to ensure feedback and accountability [24,70]. Private providers implemented different strategies to provide services to low-income and uninsured patients, such as spreading fixed costs to more paying patients, shifting to lower cadres, and providing access to government medicines and tests [25]. However, challenges such as insufficient health knowledge and skills, lack of supervision and mentorship [26], issues related to accessibility, community orientation, coordination [24], client-centredness [24,29], attitudes of health workers, and referral services [29] hindered programme effectiveness.

In Thailand, FCTs played a significant role in providing vulnerable groups with easier access to health care services. FCTs facilitated better coordination and communication across organisations, resulting in an improved transition in care [30]. Challenges in FCT implementation were related to internal communication issues among FCTs, and ambiguities in the roles of FCT managers caused concern for collaboration [30]. In Ethiopia, the community-based newborn care programme improved service utilisation for infants [31], contributing to improved child outcomes. However, challenges such as CHWs’ limited skills in correctly diagnosing key conditions in sick young infants, inadequate functional health literacy, and insufficient supportive supervision affected programme effectiveness [31].

### 3.2. Human Resources for Health

Countries implemented various workforce planning strategies to retain health care workers in PHC. While these strategies contributed to UHC by improving access and continuity of care, there were also persistent challenges. In Australia, the retention of GPs in rural and remote areas was influenced by factors such as primary income source, registrar status, hospital work and restrictions, and practice ownership [32]. However, challenges such as a lack of cultural awareness among some non-Indigenous health professionals created barriers to achieving culturally sensitive healthcare [20]. This gap in cultural competency undermines efforts to achieve equitable health care for all populations, thereby limiting progress toward UHC.

In South Africa, the retention of medical officers in PHC district health services was influenced by health facility rating, career plans, age, seniority, social and family life, teamwork, support, supervision, appreciation, and a learning environment [33]. These elements were vital in ensuring the availability of health care workers in PHC, which is essential for achieving UHC. However, confusion in the roles of nurses and doctors and power dynamics within the team impacted service delivery [34]. These challenges create gaps in the quality and continuity of care, hindering the effectiveness of PHC services and, by extension, the progress toward UHC.

In Ethiopia, almost half of the health extension workers reported job satisfaction, which was facilitated by factors such as autonomy, co-worker relationships, and recognition, and their desire to help the community, respect from the community, and achievement motivated health extension workers’ work [35]. Significant challenges remain, such as inadequate pay and benefits, limited education and career advancement opportunities, workload, and work environment that demotivate staff [35]. Moreover, less than half of health extension workers felt adequately prepared for their tasks [36]. Disparities in PHC were associated with the availability of skilled health workers per health centre between and within regions, which highlighted geographical inequalities [71].

In rural Cambodia, the retention of midwives and nurses was contributed to by factors such as optimism, appreciation of work, responsibilities, position, ability to cope with financial barriers, opportunities for professional development, job security, economic opportunities, and status in society [37]. A motivated and stable workforce in these rural areas is critical for enhancing PHC coverage and moving closer to UHC. However, persistent challenges in rural areas, such as fragmentation of service delivery, staff shortages, competition with the private sector, and shortage of medical supplies, demotivate health staff [37] and create concerns in the access to and quality of care that impede progress towards UHC.

In Nepal, Baral et al., 2013 reported persistent challenges such as vacancies in key health worker positions and a limited skill mix in PHC centres [38], limiting the capacity of the PHC system to provide universal coverage.

### 3.3. Health Information Systems

In Ethiopia, the implementation of electronic forms on smartphones was tested and considered useful for day-to-day maternal health care service delivery [40]. Key factors contributing to successful health information systems (HIS) implementation were mentoring, engagement of community and religious leaders, defined plans, and regular meetings [43]. These successes are vital for improving health service delivery and achieving UHC by ensuring that health care is accessible, coordinated, and patient-centred. However, barriers to HIS implementation in Ethiopia were significant, including poor management support, lack of accountability and supportive supervision [41], health care worker’s low digital literacy and accuracy in data entry [42], workload [43], design flaws of electronic forms and smartphones, long-term cost management [40], limited network/electricity, and absence of network [43]. Additionally, staff turnover, competing projects, lack of incentive mechanisms, institutionalisation, and ownership were challenges at the organisational level [43].

In South Africa, electronic forms/records improved patient traceability, at-home follow-ups, and timely referral by CHWs [39]. However, incorrect or incomplete personal identification led to challenges in correctly allocating patients to a ward-based outreach team [39]. These challenges impede the effectiveness of HIS, limiting its ability to fully support UHC by creating gaps in health care delivery and service coverage.

### 3.4. Essential Medicines

In South Africa, PHC played a crucial role in ensuring access to essential medicines, with the availability of at least one medicine brand per active ingredient in pharmacies that were assessed [72]. The implementation of digital stock reporting reduced stockout and increased access to medicines and vaccines [44], contributing to progress toward UHC. Although health workers in PHC were aware of the reporting system for medicines and expressed commitment to its success [46], challenges such as inadequate training, staff shortages, high turnover, and poor team communication hindered the efficient use of the digital system [46]. A combination of a community mobilisation strategy, communication initiatives, and a hotline for health care workers and patients to report drug stockouts successfully addressed stockout locally through community and health care workers’ collaboration [45]. Other factors contributing to stockouts were inadequate administration and lack of communication at PHC centres [47].

In Nepal, the impact of PHC on UHC is evident through a high dispensing rate of prescribed medicines, adequate labelling, and knowledge of medicine use among consumers [48]. However, shortages of medicines and financial burdens due to out-of-pocket expenditures [48] weaken the effectiveness of PHC in achieving UHC. Shortages of drugs and supplies in health facilities delay adequate care, resulting in referrals or the need for the patient to purchase the supplies and medicines [73].

### 3.5. Health Financing

In Thailand, universal health insurance contributed to increased PHC coverage, although some disparities remain [49]. Health insurance schemes for people with citizenship problems enabled health service providers to adapt their practices, such as having mutual agreements with hospitals to allow stateless patients to bypass primary care gatekeepers [50], improving access for stateless patients, and contributing to UHC. In Thailand, inadequate communication, unclear service guidelines, issues in residence and nationality verification procedures, and insufficient collaboration between the multiple sectors contributed to ineffectiveness in budget spending and service provision [50].

In South Africa, optimism about national health insurance reflects its potential to achieve UHC by providing equitable access to affordable health care services and collaborating between the public and private sectors [74]. However, challenges related to the government’s ability to implement national health insurance objectives, the policy relevance, and the top-down nature of implementation raised concerns about national health insurance effectiveness. Lack of collaboration, deficiencies in health care system planning and management, and the top-down nature of policymaking hindered the efficient implementation of health financing schemes [51,52].

In Ethiopia, health care utilisation increased among community-based health insurance-enrolled households, particularly benefiting the poorest [53]. In Cambodia, PHC’s role in health financing is highlighted by the pro-poor distribution of benefits from health equity funds at the primary care level in the public sector [54], improving health care access for impoverished populations.

### 3.6. Leadership and Governance

PHC governance played a vital role in addressing health equity in Australia. Strategies implemented to address the equity of access received mixed opinions regarding reaching vulnerable and marginalised families. The involvement of non-government organisations and the Aboriginal community-controlled services improved health equity by enhancing their strategies [55]. However, policy changes, budget cuts, and increased fees have compromised the comprehensiveness of the service and sustainability [55].

The Ethiopian PHC system is at a medium level in achieving UHC, including better performance in the governance domain [6]. PHC governance has shown some success in achieving UHC, with accountability mechanisms such as the community scorecard approach improving maternal and child health services, health workers’ behaviour, service availability, and reduced patient waiting time at PHCs in Ethiopia [56]. District-level leadership programmes also improved district capacity, structure and management practices, quality of care, multisectoral coordination, and trained the health workforce and functional equipment [57]. However, the sustainability and effectiveness of community scorecard implementation may face challenges over the long term [58].

In Nepal, community governance and support groups such as health committees were essential actors outside the facilities, exercising agency and influencing the implementation of reforms [59]. Health committees and service providers were aware of citizen’s charters, which was useful in providing information about the services available in the health facility [58]. However, low public awareness and poor charter implementation limited the potential to increase transparency and ensure accountability from service providers [58], posing challenges to the effectiveness of governance in achieving UHC in Nepal.

### 3.7. Community Engagement

In Australia, community engagement initiatives in PHC led to a redesign of curative services towards prevention-focused comprehensive PHC, particularly benefiting service delivery among Australian Aboriginal communities [60]. This shift enhanced service delivery and addressed health inequities, contributing to UHC.

In South Africa, community engagement has played an essential role in PHC by supporting health committees and community advisory groups that assist primary health care clinics with health promotion, day-to-day operations, and the quality delivery of health care services [63,64]. However, health committees had limited participation and decision-making and lacked influence in policy or planning budgets, which hindered the effectiveness of these committees, posing challenges to achieving UHC. The attitudes of facility managers and ward councillors, limited resources, and lack of support contributed to the limited sustainability and functioning of the health committees [63]. A lack of clarity on health committees’ roles, the skills of committee members, and limited resources impacted their effectiveness [63].

In Thailand, the migrant volunteers programme implemented in a few districts increased health care access in underserved communities. One-third of the volunteers performed their voluntary work in the community [65]. Attitudes towards the volunteer programmes were positive; the volunteers understood the programme’s benefits, and high volunteer retention was observed [65]. Yet, challenges such as community resistance to the programme due to preferences other than health care design, lack of stakeholder collaboration, lack of resources, lack of knowledge, insufficient time, communication problems, and workload among volunteers [65] limited the programme’s impact on UHC.

In Nepal, community participation strengthened PHC efforts, particularly through health facility management committees [75] and FCHVs [67]. FCHVs have played a pivotal role in delivering basic services such as distributing medicines, administering pregnancy tests, and linking the community with maternal and child health services [67], contributing to improved maternal and child health indicators. Challenges such as health committees’ lack of authority [66], geography, opportunity cost, lack of awareness, and socio-cultural discrimination were barriers to community participation [75]. FCHVs faced challenges such as inadequate incentives, a lack of education and reduced work motivation, and regional disparities in service provision [67], which limited communities’ potential in contributing to UHC. Additionally, social audits in Nepal facilitated information provision and dialogue between the community and service providers, which increased transparency, accessibility, and the quality of services, but persistent governance issues and limited authority have restricted their impact on UHC [66].

### 3.8. Multisectoral Actions

In Australia, multisectoral actions are evident in the collaboration between regional organisations (e.g., Medicare Locals/Primary Health Networks) and local governments. This collaboration was facilitated by supportive governance, resources, and public health legislation mandating a role for local governments [68], facilitating better access to health care services for marginalised communities [19], advancing UHC. Multisectoral actions between health services, community-controlled organisations, and non-government agencies were facilitated by sufficient human and financial resources, diverse backgrounds and skills, and the personal rewards that sustained commitment [69]. However, financial and time limitations and a less supportive political environment and policy for intersectoral action, including changes to primary health care [69], have posed challenges to sustaining these multisectoral actions.

## 4. Discussion

In this mixed-methods review, we synthesised multi-country evidence to provide an overall picture of the contribution of PHC systems to UHC and health status across different economies. Overall, the review shows variations in PHC systems across different economic levels. High-income countries like Australia and Belgium have expanded PHC access through telehealth and CHWs yet struggle with resource intensity and digital access disparities. The middle-income countries, South Africa and Thailand, utilised CHWs and family teams to extend care but faced skill gaps, limited support, and coordination issues. In low-income countries like Cambodia, Ethiopia, and Nepal, health equity funds and community-based volunteers expanded service reach; however, these nations grapple with shortages of supplies, staff, and consistent funding. Across all income levels, workforce retention, health information systems, and financing remain critical challenges, while community engagement and governance improve transparency but require strengthened resources and authority.

In high-income countries like Australia and Belgium, the PHC system depends on GPs, primary care teams, and centres/networks to provide various services through collaborative multidisciplinary teams [76,77]. We found that in Australia, service delivery in the community was extended through community health centres and the use of telehealth; however, using telehealth may widen the service access gap among marginalised and underserved communities [78] in resource-limited settings. These approaches ensure that services reach remote and underserved areas and contribute to improved health indicators and UHC. Despite a well-developed health system and wide PHC reach in Australia, some inequalities remain, such as health disparities faced by Aboriginal and Torres Strait Islander people and digital disparity created by telehealth approaches. These inequities could be mitigated through cohesive community engagement approaches. For example, a wide integration of A&TSIHWs to facilitate delivery of culturally safe and client-centred PHC and to mitigate the effects of a lack of cultural or self-awareness among health professionals [20], and to provide support to individuals to address digital disparities, as seen in Belgium [23]. Other challenges persist, such as financial and time limitations and a less supportive political and policy context, especially concerning changes to PHC [19,68,69].

In addition to GPs, Belgium has home care services [76]. Despite wide UHC coverage (Table 2), there are disparities in service access by socio-economically disadvantaged populations. In this review, we found that Belgium recently piloted a CHWs programme, which showed promising results in reaching disadvantaged populations [23] and contributing to reducing health disparities. This finding is coherent with findings on successful PHC service expansion and reaching disadvantaged communities through CHW implementation in South Africa, Ethiopia, and Nepal [19,25,26,27] and developing countries [79]. High-income countries may need to include communities and social determinants of health to address health inequality [80]. Integrating CHWs in health policies and programmes while addressing implementation challenges summarised in this review is a potential strategy for reducing health disparities in high-income countries, aligning with a finding from a review in high-income countries that suggested a better integration of CHWs within the broader health system [81].

Upper-middle-income countries in South Africa and Thailand have a district health system and deliver health services through health centres at the primary care level [26,30]. In this review, we found that nationwide implementation of CHWs, outreach initiatives, task shifting, and ensuring access to medicines increased UHC, provision of services and contributed to health indicators in South Africa. A similar outreach approach in communities implemented through FCTs increased access to PHC services in Thailand. FCTs comprise multidisciplinary teams available in districts, subdistricts, and communities. At the community level, FCT comprises village health volunteers, local organisations, community leaders, and carers [30].

In Ethiopia, community-based newborn care programmes to expand community services and link to child services increased access to newborn and child health services, ultimately reducing child mortality. The PHC system in Ethiopia includes health posts, health centres, and district hospitals. While health posts are the first point of contact with the health system, CHWs support health activities at the community level [35]. However, ensuring adequate knowledge and skills of CHWs is crucial to delivering appropriate and safe newborn care.

Retention of a skilled and motivated workforce is required for effective service delivery. In this review, some factors motivating the retention of health workers were common between countries. Still, specific factors also influenced retention in each country and by the type of health workforce. Nevertheless, policymakers and programme implementers must promote retention factors and address health workers’ challenges to ensure effective service delivery. Across countries, job satisfaction, financial security, and autonomy contributed to workforce retention. In Australia, income source and position were important for the health workforce, but there were some barriers related to cultural awareness in providing culturally sensitive health care [20]. In South Africa, the status of health facilities, teamwork, and learning environments facilitated retention. In Cambodia [37] and Ethiopia [35], societal recognition and respect were necessary for health workers to continue their work [35]. A lack of professional opportunities, inadequate pay, and a sub-optimal work environment in Ethiopia, and staff shortages in Cambodia and Nepal created challenges in service delivery [37,38]. These barriers may widen the health inequity gap and slow progress towards the SDGs and achieving UHC [82].

Very limited studies on HIS were identified in this review. Existing evidence suggests HIS contributes to improvement in PHC service delivery and utilisation [83], and our review supports this association. Although there were some challenges in South Africa and Ethiopia, using electronic records in PHC services facilitated service delivery. Addressing infrastructure and resource constraints is paramount for successful HIS implementation and sustainability across different economic levels [21,40,43]. In low- and lower-middle-income countries, additional organisational challenges such as lack of management support, accountability, limited institutionalisation, and ownership need to be addressed [43].

Regarding health financing, different financing schemes were implemented to promote equity in access to health care services, resulting in increased health care utilisation. However, health financing initiatives had varying impacts on improving equitable PHC access in Thailand, South Africa, Ethiopia, and Cambodia [49,53,54]. Common challenges such as unclear service guidelines, legal restrictions in Thailand [49,50], lack of collaboration, deficiencies in health care system planning and management, and the top-down nature of policymaking in South Africa hindered the efficient implementation of health financing schemes. The fragmentations of health financing have implications for the low availability of medicines in PHC centres [48,73] and out-of-pocket medical expenditures for medicine and PHC services [48,49]. The extent of patients’ financial burdens due to out-of-pocket expenditures may vary between countries based on health care financing mechanisms.

Several governance approaches were utilised to improve PHC in Australia, Ethiopia, and Nepal [6,55,56,57,58,59]. Involvement of non-government organisations and the Aboriginal community in Australia, leadership programmes and community scorecard approaches in Ethiopia, and a citizen’s charter in Nepal were some approaches used. Governance was also facilitated through community engagement initiatives such as community advisory groups in South Africa, health facility committees, and social audits in Nepal. Although community involvement in all PHC services planning, design, delivery, and evaluation stages was abysmal, these approaches effectively governed PHC, facilitating social accountability by engaging with health providers to enable dialogue, enhance transparency, and strengthen community partnerships [64,66,84].

In Australia, South Africa, Thailand, and Nepal, community engagement efforts must be more cohesive to improve PHC service delivery and the achievement of UHC. Although community engagement in low-income countries is more common and implemented nationally, such as in Nepal, participation may sometimes be tokenistic only to fulfil administrative criteria with less interest and influence in policy or planning budgets for local PHCs [84]. The wider socio-economic context, culture, lack of community support, and organisational issues often influence community engagement, aligning with findings from a recent scoping review on community engagement in PHC [14]. In high-income countries such as Australia and Belgium, the traditional focus of PHC may need to shift from a cure and medical perspective to include communities and social determinants of health [80]. Integrating CHWs in health policies and programmes while addressing implementation challenges summarised in this review is a potential strategy for reducing health disparities in high-income countries. A similar finding from a review in high-income countries suggested a better integration of CHWs within the broader health system [81].

Given multisectoral action is a fundamental tenet of PHC, it was surprising to find a limited number of studies from the seven countries. In Australia, multisectoral actions between health services, government, and communities were facilitated by adequate human and financial resources, diverse skills, and personal rewards sustaining commitment [19,68,69]. Multisectoral actions contribute to PHC by enabling strategic policy directions, optimising spillover effects of other sectors such as water, sanitation and hygiene, information technology, and infrastructure on health, supporting the provision and utilisation of PHC, and ultimately contributing to improved health outcomes [13].

### 4.1. Implications for Programs and Policies

In high-income countries, integration of CHWs within the broader health system can help address health disparities observed among specific populations. This may involve initiatives like training A&TSIHWs in Australia or similar community engagement strategies tailored to specific contexts. Using digital health technologies like telehealth to enhance service delivery must ensure that its adoption does not widen the service access gap among marginalised and underserved communities. Ensuring culturally sensitive and community-centred PHC delivery through community engagement and addressing social determinants of health could reduce health inequality and improve the population’s overall health.

Middle-income countries should strengthen CHWs and outreach programmes, which are vital for delivering services to disadvantaged and vulnerable populations. Community engagement efforts should be enhanced to ensure culturally safe and client-centred PHC delivery. Countries must address challenges related to workforce retention and skill shortages to ensure effective primary health care delivery and reach disadvantaged and vulnerable populations.

Low-income countries need to invest in developing and strengthening HIS to improve PHC delivery and utilisation. Efforts to promote equity in access to health care services through different financing schemes should be intensified by addressing challenges such as infrastructure, resource shortages, and financial barriers to access. Improving governance approaches and community engagement initiatives such as health facility committees and social audits can enhance transparency, accountability, and community participation in PHC.

### 4.2. Limitations

We used a narrative approach to synthesise evidence on PHC systems’ contribution to selected health indicators at the population level. Therefore, we cannot infer direct associations between the PHC systems and health indicators in this review. The literature synthesis for three economic levels is based on only seven countries. Furthermore, we could not synthesise evidence for each country on all eight domains assessed due to limited published literature. Another limitation of this review is the purposeful selection of countries. We also selected studies published in the English language only. These may have led to some selection bias and influenced the results. Future reviews on PHC systems could focus on a global setting and include studies published in other languages. Another methodological limitation is the absence of quality assessment of the literature included in this review. We followed a scoping review approach to scope the literature on PHC systems, as our aim was not to generate a critically appraised answer to a specific question. However, we provide some recommendations in this review, which should be interpreted with caution as there is an inherent risk of bias associated with the primary literature included in this review.

## 5. Conclusions

Countries implemented strategies to strengthen their PHC systems towards UHC, particularly for remote and vulnerable populations, through targeted initiatives such as telehealth, CHWs, and multisectoral partnerships. However, persistent challenges in workforce retention, digital infrastructure, healthcare financing, and governance hinder their full effectiveness. Community engagement and multisectoral actions contributed to service improvements, but governance issues, resource constraints, and inequities persisted across regions. Community engagement and CHW programmes could be integrated in all countries to extend services to hard-to-reach populations. Countries should invest in developing and strengthening digital health technologies, including HIS and telehealth, for effective PHC service delivery. Health financing initiatives in the upper-middle- and low-income countries reviewed in this study may benefit from implementation research to identify and mitigate the challenges preventing effective implementation and scaling up. Despite the varying contexts of the reviewed countries, leveraging successful strategies and addressing the challenges synthesised in this review can lead countries closer to achieving the goal of UHC and improving health outcomes for all.

## Figures and Tables

**Table 1:** Key health indicators and universal health coverage in the seven countries.

Country	Health Indicators			Universal Health Coverage Index			
	Life Expectancy in Years (2021)	Maternal Deaths per 100,000 Live Births (2020)	Child Mortality Rate per 1000 Live Births (2021)	UHCI	UHCI for Reproductive, Maternal, and Child Health	UHCI for Infectious Diseases	UHCI for Non-Communicable Diseases
Belgium	81.9	5	4	86	94	91	72
Australia	84.5	3	4	87	91	90	72
South Africa	62.3	127	33	71	76	69	61
Thailand	78.7	29	8	82	89	84	67
Cambodia	69.6	218	25	58	74	65	64
Ethiopia	65.0	267	47	35	48	29	52
Nepal	68.4	174	27	54	78	62	46

Source: Our World in Data [17]. Maternal deaths—the number of women who die from pregnancy-related causes while pregnant or within 42 days of pregnancy termination. Child mortality rate—deaths per 1000 live births.

**Table 2:** Summary of successes and challenges of PHC systems.

Domains	Countries	Successes	Challenges
Service delivery	Australia [18,19,20]	Telehealth to reach remote communities. Community health centres, people-centred Aboriginal and/or Torres Strait Islander health workers to expand access among Aboriginal people (A&TSIHWs).	Telehealth was resource intensive, increased staff workload, and may exclude populations not owning smartphones. Limited range and quality of services in remote areas [21,22].
	Belgium [23]	Community health workers (CHWs) and outreach initiatives to reach vulnerable communities.	
	South Africa [24,25,26,27,28]	CHWs and outreach initiatives (nationwide but implementation varied) to reach communities. Task shifting, providing access to government medicines and tests to provide services to low-income and uninsured patients.	CHWs’ lack of health knowledge, skills, supervision, and mentoring. Accessibility, community orientation, coordination, client-centredness, attitudes of health workers, and referral services [24,26,29].
	Thailand [30]	Family and community teams (FCTs) providing health care to vulnerable groups.	Internal communication among FCTs and ambiguities in the roles of FCT managers causing a concern for collaboration.
	Ethiopia [31]	Community-based newborn care programme to expand services in communities and link to child services.	Inadequate knowledge, skills.
Workforce	Australia [32]	Income source, registrar status, hospital work and restrictions, and practice ownership influenced general practitioner (GP) retention in remote areas.	Lack of cultural awareness created barriers to culturally sensitive health care [20]
	South Africa [33]	Health facility rating, career plans, seniority, social and family life, teamwork, support, supervision, appreciation, and a learning environment facilitated the retention of medical officers.	Issues in teamwork due to power dynamics [34]
	Ethiopia [35]	Job satisfaction, financial security, societal recognition, and autonomy influenced the retention of medical officers.	Lack of professional opportunities, inadequate pay, unsupportive work environment [35,36]
	Cambodia [37]	Appreciation of work, position, professional development opportunities, job security, and financial opportunities were important for nurses and midwives.	Fragmentation of service delivery, staff shortages, competition with the private sector, and shortage of medical supplies.
	Nepal [38]		Persistent vacant positions and limited skill mix.
Information system	South Africa [39]	Use of electronic records for patient follow-ups and referrals.	Incomplete personal information for tracing.
	Ethiopia [39,40]	Electronic forms used to facilitate service delivery.	Lack of infrastructure, resources, management support, limited network/electricity, accountability, limited institutionalisation, and ownership [39,41,42,43]
Essential medicines	South Africa [44,45]	Digital stock reporting for medicines and vaccines reduced stockouts and increased access. Community mobilisation, communication, reporting drug stockouts by community-addressed stockout.	Inadequate training, staff shortages, high turnover, poor team communication [46,47].
	Nepal [48]	Adequate labelling and knowledge of medicine use.	Medicine shortage, out-of-pocket expenditures.
Financing	Thailand [49,50]	Universal health insurance: health insurance for people with citizenship problems expanded service access to migrants/people without citizenship.	Some disparities in access, unclear service guidelines, and legal restrictions.
	South Africa [51]	Policymakers were optimistic about national health insurance.	Lack of collaboration, deficiencies in health care system planning and management, and top-down nature of policymaking. Out-of-pocket expenditures [51,52]
	Ethiopia [53]	Community-based health insurance benefitted the poorest.	
	Cambodia [54]	Health equity funds targeted the poorest.	
Governance	Australia [55]	The involvement of non-government organisations and the Aboriginal community-controlled services improved health equity.	Budget cuts led to increased fees and a loss of comprehensiveness in service.
	Ethiopia [56,57]	Social accountability mechanisms improved health service quality and delivery.	
	Nepal [58,59]	Health committees and citizen charters to implement reforms and inform communities about health services.	Low public awareness, poor implementation of citizen’s charters [58]
Community engagement	Australia [60]	Community engagement initiative in services targeted among Aboriginal peoples.	
	South Africa [28,61,62]	Health committees’ community advisory groups assisting PHCs with service delivery. CHWs.	Health committees had limited participation, and decision-making lacked influence in policy or planning budgets. Lack of clarity on health committees’ roles, skills of committee members, and limited resources. Gender issues, poor community understanding of CHWs’ roles, environmental challenges, lack of permanent employment, limited resources, and low wages [28,61,62,63,64]
	Thailand [65]	Migrant volunteer programmes increased health care access in underserved communities.	Community resistance, poor stakeholder collaboration, low resources, unsupportive health care system.
	Nepal [66,67]	Social audits, health committees, and female community health volunteers (FCHVs) to reach communities.	Weak provision of sanctions lack authority to implement the action plan or participate in the management of service providers.
Multisectoral actions	Australia [19,68,69]	Collaboration between health service, government, community-controlled organisations, and non-government agencies to expand services among marginalised communities.	Resource limitations, unsupportive political and policy context, changes to PHC.

## Data Availability

We did not generate or analyse data for this study, and all information generated during this study is included in this published article and its supplementary information files.

## Supplementary Materials

The following supporting information can be downloaded at https://www.mdpi.com/article/10.3390/ijerph21121601/s1, Document S1: Search strategy and included studies. Table S1: List of studies included in the review.
