# Supplementary material for: Primary Health Care Systems and Their Contribution to Universal Health Coverage and Improved Health Status in Seven Countries: An Explanatory Mixed-Methods Review
Source: Int J Environ Res Public Health. 2024 Nov 30;21(12):1601. doi: 10.3390/ijerph21121601 (PMC11675242; doi:10.3390/ijerph21121601)
Supplement: Supplementary file 1 [file ijerph-21-01601-s001.zip › ijerph-3236830-supplementary.pdf]

Document S1

Search Strategy

1. **Strategy**

Primary health care

"Primary health care" OR "Primary care" OR "Primary healthcare"

Six building blocks:

"service delivery" OR "health service delivery" OR

"health workforce" OR "workforce" OR "health work*" OR "healthcare work*" OR

"health information system" OR "information system" OR

"essential medicines" OR "medicines" OR "essential drugs" OR "essential vaccin*" OR "essential diagnostic*" OR

"health financing" OR "health insurance" OR "social protection" OR "social health insurance" OR "healthcare financing" OR

"governance for health" OR "health governance" OR "health system governance" OR "health leadership" OR

Other relevant terminologies: "Health system strengthening" OR "Health system framework" OR "health system building blocks" OR

Community engagement: "User involvement" OR "User participation" OR "User contribution" OR "Community collaboration" OR "Community participation" OR "Community representative" OR "Community engagement" OR "Community input" OR "Community led" OR "Community involvement" OR "Lay representative" OR "Lay perspective" OR "Lay perception" OR "Lay involvement" OR "Lay participation" OR "Consumer participation" OR "consumer involvement" OR "consumer groups" OR "consumer driven" OR "consumer engagement" OR "patient perspective" OR "patient involvement" OR "patient participation" OR "patient representative" OR "patient engagement" OR "citizen participation" OR "citizen involvement" OR "citizen engagement" OR "citizen deliberation" OR

Multisectoral actions: "multisectoralism" OR "intersectorality" OR "coordination" OR "collaboration" OR "multisectoral*" OR "intersectoral*" OR "multisectoral action" OR "intersectoral coordination" OR "intersectoral action*"

Countries:

"Belgium" OR "Australia" OR "South Africa" OR "Thailand" OR "Cambodia" OR "Ethiopia" OR "Nepal"

2. **Search execution**

Database 1: Pubmed

Hits 1075

((((("primary health care" [Title/Abstract] OR "primary care" [Title/Abstract]) OR "primary healthcare" [Title/Abstract]) AND (("service delivery" [Title/Abstract]) OR ("health service delivery" [Title/Abstract]) OR (workforce [Title/Abstract]) OR ("health workforce" [Title/Abstract]) OR ("health* work*" [Title/Abstract]) OR ("information system" [Title/Abstract]) OR ("health information system" [Title/Abstract]) OR ("essential medicines" [Title/Abstract]) OR (medicines [Title/Abstract]) OR ("essential drug*" [Title/Abstract]) OR (financing[Title/Abstract]) OR ("health* financing" [Title/Abstract]) OR ("health insurance" [Title/Abstract]) OR ("financial protection" [Title/Abstract]) OR ("social* protection" [Title/Abstract]) OR (governance[Title/Abstract]) OR ("health governance" [Title/Abstract]) OR ("health* governance" [Title/Abstract]) OR ("governance for health" [Title/Abstract]) OR ("health leadership" [Title/Abstract]) OR ("Health system strengthening" [Title/Abstract]) OR ("Health system framework" [Title/Abstract]) OR ("health system building blocks" [Title/Abstract]) OR ("User involvement" [Title/Abstract]) OR ("User participation"[Title/Abstract]) OR ("User contribution"[Title/Abstract]) OR ("Community collaboration"[Title/Abstract]) OR ("Community participation"[Title/Abstract]) OR ("Community representative"[Title/Abstract]) OR ("Community engagement"[Title/Abstract]) OR ("Community input"[Title/Abstract]) OR ("Community led"[Title/Abstract]) OR ("Community involvement"[Title/Abstract]) OR ("Lay representative"[Title/Abstract]) OR ("Lay perspective"[Title/Abstract]) OR ("Lay perception"[Title/Abstract]) OR ("Lay involvement"[Title/Abstract]) OR ("Lay participation"[Title/Abstract]) OR ("Consumer participation"[Title/Abstract]) OR ("consumer involvement"[Title/Abstract]) OR ("consumer groups"[Title/Abstract]) OR ("consumer driven"[Title/Abstract]) OR ("consumer engagement"[Title/Abstract]) OR ("patient perspective"[Title/Abstract]) OR ("patient involvement"[Title/Abstract]) OR ("patient participation"[Title/Abstract]) OR ("patient representative"[Title/Abstract]) OR ("patient engagement"[Title/Abstract]) OR ("citizen participation"[Title/Abstract]) OR ("citizen involvement" [Title/Abstract]) OR ("citizen engagement"[Title/Abstract]) OR ("citizen deliberation"[Title/Abstract])))) AND ((Belgium[Title/Abstract]) OR (Australia[Title/Abstract]) OR (Thailand[Title/Abstract]) OR (South Africa[Title/Abstract]) OR (Cambodia[Title/Abstract]) OR (Ethiopia[Title/Abstract]) OR (Nepal[Title/Abstract])))

Limits: Year 2000 onwards, Published in English, Human studies

Database 2: Scopus

Hits 2285

TITLE-ABS-KEY ("Primary health care" OR "Primary care" OR "Primary healthcare") AND TITLE-ABS-KEY ("service delivery" OR "health service delivery" OR "health workforce" OR "workforce" OR "health work*" OR " healthcare work* " OR " health AND information AND system " OR " information AND system " OR " essential AND medicines " OR " medicines " OR " essential AND drugs " OR " essential AND vaccin* " OR " essential AND diagnostic* " OR " health AND financing " OR " health AND insurance " OR " social AND protection " OR " social AND health AND insurance " OR " healthcare AND financing " OR " governance AND for AND health " OR " health AND

governance " OR " health AND system AND governance " OR " health AND leadership " OR " health AND system AND strengthening " OR " health AND system AND framework " OR " health AND system AND building AND blocks " OR " user AND involvement " OR " user AND participation " OR " user AND contribution " OR " community AND collaboration " OR " community AND participation " OR " community AND representative " OR " community AND engagement " OR " community AND input " OR " community AND led " OR " community AND involvement " OR " lay AND representative " OR " lay AND perspective " OR " lay AND perception " OR " lay AND involvement " OR " lay AND participation " OR " consumer AND participation " OR " consumer AND involvement " OR " consumer AND groups " OR " consumer AND driven " OR " consumer AND engagement " OR " patient AND perspective " OR " patient AND involvement " OR " patient AND participation " OR " patient AND representative " OR " patient AND engagement " OR " citizen AND participation " OR " citizen AND involvement " OR " citizen AND engagement " OR " citizen AND deliberation " OR " multisectoralism " OR " intersectorality " OR " coordination " OR " collaboration " OR " multisectoral* " OR " intersectoral* " OR " multisectoral AND action " OR " intersectoral AND coordination " OR " intersectoral AND action*") AND TITLE-ABS-KEY ("belgium" OR "australia" OR "south AND africa" OR "thailand" OR "cambodia" OR "ethiopia" OR "nepal")

Limits: Year 2000 onwards, Published in English, Human studies

Database 3: Embase

Hits 1947

'primary health care'/exp OR 'primary health care' OR 'primary care'/exp OR 'primary care' OR 'primary healthcare':ab AND 'service delivery':ab OR 'health service delivery':ab OR 'health workforce':ab OR 'workforce':ab OR 'health work*':ab OR 'healthcare work*':ab OR 'health information system':ab OR 'information system':ab OR 'essential medicines':ab OR 'medicines':ab OR 'essential drugs':ab OR 'essential vaccin*':ab OR 'essential diagnostic*':ab OR 'health financing':ab OR 'health insurance':ab OR 'social protection':ab OR 'social health insurance':ab OR 'healthcare financing':ab OR 'governance for health':ab OR 'health governance':ab OR 'health system governance':ab OR 'health leadership':ab OR 'health system strengthening':ab OR 'health system framework':ab OR 'health system building blocks':ab OR 'user involvement':ab OR 'user participation':ab OR 'user contribution':ab OR 'community collaboration':ab OR 'community participation':ab OR 'community representative':ab OR 'community engagement':ab OR 'community input':ab OR 'community led':ab OR 'community involvement':ab OR 'lay representative':ab OR 'lay perspective':ab OR 'lay perception':ab OR 'lay involvement':ab OR 'lay participation':ab OR 'consumer participation':ab OR 'consumer involvement':ab OR 'consumer groups':ab OR 'consumer driven':ab OR 'consumer engagement':ab OR 'patient perspective':ab OR 'patient involvement':ab OR 'patient participation':ab OR 'patient representative':ab OR 'patient engagement':ab OR 'citizen participation':ab OR 'citizen involvement':ab OR 'citizen engagement':ab OR 'citizen deliberation':ab OR 'multisectoralism':ab OR 'intersectorality':ab OR 'coordination':ab OR 'collaboration':ab OR 'multisectoral*':ab OR 'intersectoral*':ab OR 'multisectoral action':ab OR 'intersectoral coordination':ab OR 'intersectoral action*':ab AND 'belgium':ab OR 'australia':ab OR 'south and africa':ab OR 'thailand':ab OR 'cambodia':ab OR 'ethiopia':ab OR 'nepal':ab

Database 4: APApsycinfo

Hits 231

AB (“Primary health care” OR “Primary care” OR “Primary healthcare”) AND AB (“service delivery” OR “health service delivery” OR “health workforce” OR “workforce” OR “health work*”

OR “healthcare work*” OR “health information system” OR “information system” OR “essential medicines” OR “medicines” OR “essential drugs” OR “essential vaccin*” OR “essential diagnostic*” OR “health financing” OR “health insurance” OR “social protection” OR “social health insurance” OR “healthcare financing” OR “governance for health” OR “health governance” OR “health system governance” OR “health leadership” OR “Health system strengthening” OR “Health system framework” OR “health system building blocks” OR "User involvement" OR "User participation" OR "User contribution" OR "Community collaboration" OR "Community participation" OR "Community representative" OR "Community engagement" OR "Community input" OR "Community led" OR "Community involvement" OR "Lay representative" OR "Lay perspective" OR "Lay perception" OR "Lay involvement" OR "Lay participation" OR "Consumer participation" OR "consumer involvement" OR "consumer groups" OR "consumer driven" OR "consumer engagement" OR "patient perspective" OR "patient involvement" OR "patient participation" OR "patient representative" OR "patient engagement" OR "citizen participation" OR "citizen involvement" OR "citizen engagement" OR "citizen deliberation" OR “multisectoralism” OR “intersectorality” OR “coordination” OR “collaboration” OR “multisectoral*” OR “intersectoral*” OR “multisectoral action” OR “intersectoral coordination” OR “intersectoral action*”) AND AB (“Belgium” OR “Australia” OR “South Africa” OR “Thailand” OR “Cambodia” OR “Ethiopia” OR “Nepal”)

Limits: Year 2000 onwards, Published in English

Database 5: CINAHL

Hits 874

AB (“Primary health care” OR “Primary care” OR “Primary healthcare”) AND AB ("service delivery" OR "health service delivery" OR workforce OR "health workforce" OR "health* work*" OR "information system" OR "health information system" OR "essential medicines" OR medicines OR "essential drug*" OR financing OR "health* financing" OR "health insurance" OR "financial protection" OR "social* protection" OR governance OR "health governance" OR "health* governance" OR "governance for health" OR "health leadership" OR "Health system strengthening" OR "Health system framework" OR "health system building blocks" OR "User involvement" OR "User participation" OR "User contribution" OR "Community collaboration" OR "Community participation" OR "Community representative" OR "Community engagement" OR "Community input" OR "Community led" OR "Community involvement" OR "Lay representative" OR "Lay perspective" OR "Lay perception" OR "Lay involvement" OR "Lay participation" OR "Consumer participation" OR "consumer involvement" OR "consumer groups" OR "consumer driven" OR "consumer engagement" OR "patient perspective" OR "patient involvement" OR "patient participation" OR "patient representative" OR "patient engagement" OR "citizen participation" OR "citizen involvement" OR "citizen engagement" OR "citizen deliberation") AND AB (“Belgium” OR “Australia” OR “South Africa” OR “Thailand” OR “Cambodia” OR “Ethiopia” OR “Nepal”)

Limits: Year 2000 onwards, Published in English

Database 6: Cochrane Library

Hits 125

(“Primary health care”):ti,ab,kw OR (“Primary care”):ti,ab,kw OR (“Primary healthcare”):ti,ab,kw

AND

("service delivery"):ti,ab,kw OR ("health service delivery"):ti,ab,kw OR (workforce):ti,ab,kw OR ("health workforce"):ti,ab,kw OR ("health* work*"):ti,ab,kw OR ("information system"):ti,ab,kw OR ("health information system"):ti,ab,kw OR ("essential medicines"):ti,ab,kw OR (medicines):ti,ab,kw OR ("essential drug*"):ti,ab,kw OR (financing):ti,ab,kw OR ("health* financing"):ti,ab,kw OR ("health insurance"):ti,ab,kw OR ("financial protection"):ti,ab,kw OR ("social* protection"):ti,ab,kw OR (governance):ti,ab,kw OR ("health governance"):ti,ab,kw OR ("health* governance"):ti,ab,kw OR ("governance for health"):ti,ab,kw OR ("health leadership"):ti,ab,kw OR ("Health system strengthening"):ti,ab,kw OR ("Health system framework"):ti,ab,kw OR ("health system building blocks"):ti,ab,kw OR ("User involvement"):ti,ab,kw OR ("User participation"):ti,ab,kw OR ("User contribution"):ti,ab,kw OR ("Community collaboration"):ti,ab,kw OR ("Community participation"):ti,ab,kw OR ("Community representative"):ti,ab,kw OR ("Community engagement"):ti,ab,kw OR ("Community input"):ti,ab,kw OR ("Community led"):ti,ab,kw OR ("Community involvement"):ti,ab,kw OR ("Lay representative"):ti,ab,kw OR ("Lay perspective"):ti,ab,kw OR ("Lay perception"):ti,ab,kw OR ("Lay involvement"):ti,ab,kw OR ("Lay participation"):ti,ab,kw OR ("Consumer participation"):ti,ab,kw OR ("consumer involvement"):ti,ab,kw OR ("consumer groups"):ti,ab,kw OR ("consumer driven"):ti,ab,kw OR ("consumer engagement"):ti,ab,kw OR ("patient perspective"):ti,ab,kw OR ("patient involvement"):ti,ab,kw OR ("patient participation"):ti,ab,kw OR ("patient representative"):ti,ab,kw OR ("patient engagement"):ti,ab,kw OR ("citizen participation"):ti,ab,kw OR ("citizen involvement"):ti,ab,kw OR ("citizen engagement"):ti,ab,kw OR ("citizen deliberation"):ti,ab,kw

AND

(Belgium):ti,ab,kw OR (Australia):ti,ab,kw OR (Thailand):ti,ab,kw OR (South Africa):ti,ab,kw OR (Cambodia):ti,ab,kw OR (Ethiopia):ti,ab,kw OR (Nepal):ti,ab,kw

Limits: Year 2000 onwards, Published in English

Table S1. List of studies included in the review.

	Author, Year	Country	Title	Aim	Study Design	Findings
1	Mathew et al, 2023 [1]	Australia	Telehealth in remote Australia: a supplementary tool or an alternative model of care replacing face-to-face consultations?	Examine the perspectives of remote PHC staff in regard to the use, drivers, and limitations of conducting specialist and PHC consultations via telehealth.	Qualitative interviews and discussion groups	Reduced need to travel, telehealth functioned best when there was a pre-established relationship between the patient and the health care provider, patients who had good knowledge of their personal health, spoke English and had access to and familiarity with digital technology.<br>Resource intensive, increasing staff workload, an interpreter for translation services.<br>A useful supplementary tool, and not a stand-alone service model replacing face-to-face interactions.
2	Ashley et al, 2023 [2]	Australia	Telehealth's future in Australian primary health care: a qualitative study exploring lessons learnt from the COVID-19 pandemic	Investigate GPs', registered nurses' , nurse practitioners', and allied health clinicians perceptions of the sustainability of telehealth in PHC post-pandemic	Qualitative	A hybrid approach, combining face to face and telehealth, would be preferable. Support for health professionals is required to sustain telehealth services, including training to address knowledge gaps and develop telehealth skills.
3	Nolan-Isles et al, 2021 [3]	Australia	Enablers and barriers to accessing healthcare services for aboriginal people in new south wales, australia	Investigate the barriers and enablers experienced by Aboriginal people across the life course in accessing primary, specialist and allied healthcare services in regional and remote Australia	Qualitative semi-structured interviews	Six themes identified: (1) Improved coordination of healthcare services; (2) Better communication between services and patients; (3) Trust in services and cultural safety; (4) Importance of prioritizing health services by Aboriginal people; (5) Importance of reliable, affordable and sustainable services; (6) Distance and transport availability. These themes were often present as both barriers and enablers to healthcare access for Aboriginal people.

4	Butler et al, 2021 [4]	Australia	Examining area-level variation in service organisation and delivery across the breadth of primary healthcare. Usefulness of measures constructed from routine data	Constructing area-level measures of PHC service organisation and delivery in Australia, covering access, comprehensiveness, and coordination, to explore their utility in assessing area-level variation in the PHC system.	Secondary data analysis	PHC service delivery varied geographically within cities and more remote locations. Areas in major cities were more accessible , while in remote areas, services were more comprehensive and coordinated. In disadvantaged areas of major cities, there were fewer GPs , services were more affordable , a greater proportion were after-hours and for chronic disease care but fewer for preventive care. Patterns were similar in regional locations, other than disadvantaged areas had less after-hours care.
5	Topp et al, 2021 [5]	Australia	Unique knowledge, unique skills, unique role: Aboriginal and Torres Strait Islander Health Workers in Queensland, Australia	To map the unique purpose, functions and skills required, of A&TSIHWs	Qualitative	The A&TSIHW role is multifaceted and comprises three core functions: health promotion, clinical service and cultural brokerage. A&TSIHWs hold both biomedical and Aboriginal and Torres Strait Islander knowledges and apply them as appropriate to shape service delivery to Aboriginal and Torres Strait Islander clients. One of the only mechanisms available in frontline services through which active adaptations can be made to account for the different knowledge systems that shape Aboriginal and Torres Strait Islander peoples' understanding of health.
6	Freeman et al, 2016 [6]	Australia	Revisiting the ability of Australian primary healthcare services to respond to health inequity	Examine the effect of the changes in PHC over 5 years (2009–2013) on services' ability to achieve equity of access, facilitate wider health care and address social determinants of health inequities.	Qualitative	At the four state government services, seven of 10 previously identified strategies for equity were compromised or reduced in some way as a result of the changing policy environment. There was a mix of positive and negative changes at the non-government organisation. The community controlled service increased their breadth of strategies used to address health equity.
7	Reeve et al, 2015 [7]	Australia	Community participation in health service reform: the	Describe the reorientation of a remote primary health-care service, in the Kimberley region of	Mixed methods: literature review,	A community-led partnership providing sustainable health services resulted in increased access to primary health care. Three major factors facilitated the change- strong local community participation, strong local

			development of an innovative remote Aboriginal primary health-care service	Australia, its impact on access to services and the factors instrumental in bringing about change.	qualitative and quantitative studies	community and health service vision aligning with the goals underpinning State and Commonwealth government health policies, partnership instituted changes in service delivery by making significant structural changes to how, where and who delivered service through engaging staff and consistently reinforcing the changes.
8	Anaf et al, 2014 [8]	Australia	Factors shaping intersectoral action in primary health care services	Identify factors that facilitate and constrain intersectoral action in PHC services in six sites in South Australia and the Northern Territory	Descriptive case studies	Factors facilitating intersectoral action included sufficient human and financial resources, diverse backgrounds and skills and the personal rewards that sustain commitment. Key constraining factors were financial and time limitations, and a political and policy context which has become less supportive of intersectoral action; including changes to primary health care.Diversity in backgrounds and skills of individuals from collaborating organisations was regarded as a key advantage to intersectoral action in health.
9	Russell et al, 2012 [9]	Australia	What factors contribute most to the retention of general practitioners in rural and remote areas?	Measure the strength and contribution of significant factors associated with the retention of Australian GPs	Secondary data analysis	Factors associated with the retention of rural and remote GPs were: primary income source, registrar status, hospital work and restrictions on practice location, practice ownership; less important factors included geographic location, procedural skills, annual leave, workload and practice size. Findings quantify a range of financial and economic, professional and organisational, and geographic factors contributing to the retention of rural GPs.
10	Javanprast et al, 2019 [10]	Australia	Collaborative population health planning between Australian primary health care organisations and	Examine the strength and extent of collaborations between primary health careorganisations and local government in population health planning	Mixed-methods: online surveys, interviews and a review of reports	Medicare locals/Primary healh networks reported limited time and financial support for collaboration with local government. Organisational capacity and resources, supportive governance and public health legislation mandating a role for local governments were critical to collaborative planning

			local government: lost opportunity			
11	Masquillier & Cosaert, 2023 [11]	Belgium	Facilitating access to primary care for people living in socio-economically vulnerable circumstances in Belgium through community health workers: towards a conceptual model	Explore the ways in which CHWs support people living in socio-economically vulnerable circumstances in their access to primary care	Qualitative	CHW outreach reach vulnerable people such as a low level of education; being at risk of poverty and severe material deprivation; having no or limited knowledge of the local language; being of an older age; having a limited social network; being a newcomer; having no legal residence status or being in request for international protection; having a physical disability; living with a mental illness; and/or being homelessexperiencing complex care needs, facing several barriers that interrupt the continuum of access to primary care.
12	Asante et al, 2019 [12]	Cambodia	Who benefits from healthcare spending in Cambodia? Evidence for a universal health coverage policy	Assesses how benefits from healthcare spending are distributed across socioeconomic groups in Cambodia.	Secondary data analysis	Health financing in Cambodia appears to benefit the poor more than the rich but a significant proportion of spending remains in the private sector which is largely pro-rich. Public hospital outpatient benefits are quite evenly distributed across all wealth quintiles, although the concentration index of 0.058 suggests a moderately pro-poor distribution. Benefits for public hospital inpatient care are substantially pro-poor. The private sector was significantly skewed towards the richest quintile.
13	Chhea et al, 2010 [13]	Cambodia	Health worker effectiveness and retention in rural Cambodia	In this article the strategies of and barriers met by health workers who remain in rural areas and deliver public health services are elucidated.	Qualitative	Primary healthcare service delivery in rural Cambodia was reliant on the retention of mid-level of health staff, primarily midwives and nurses. The interaction between institutional factors and personal factors was crucial for effectiveness of health staff retention in rural Cambodia.

						Institutional factors- fragmentation of service delivery and structure, limited capacity and shortage of high-qualified health staff, competition with the private sector, and shortage of medical supplies demotivated staff. Optimism, appreciation of work, ability to cope with financial barriers, opportunities for professional development, job security, financial opportunities, and status in society were important personal factors.
14	Yitbarek et al, 2023 [14]	Ethiopia	Capacity of the Ethiopian primary health care system to achieve universal health coverage: a primary health care progression approach	Measure the capacity of PHC in Ethiopia to assess how the PHC system is progressing towards an optimized capacity for delivery of effective PHC.	Mixed-methods: secondary data analysis, document review, key-informant interviews	The Ethiopian PHC system is at a medium level in achieving UHC, including better performance in the governance domain. The challenges related to major inputs coupled with data-quality problems reduced the capacity of the PHC system at the local level.
15	Hailemariam et al, 2023 [15]	Ethiopia	Individual and contextual level enablers and barriers determining electronic community health information system implementation in northwest Ethiopia	Explore individual and contextual-level enablers and barriers determining eCHIS implementation.	Qualitative	Implementers valued the eCHIS program but implementation was impacted by the heavy workload, limited or absent network and electricity, staff turnover, presence of competing projects, and lack of incentive mechanisms, lack of institutionalization and ownership. Limited digital literacy, older age, lack of peer-to-peer support, and limited self-expectancy posed challenges to the implementation.
16	Ejigu et al, 2023 [16]	Ethiopia	Motivation and job satisfaction of community health workers in Ethiopia: a mixed-methods approach.	The aim of this study was to assess the HEWs' motivation and job satisfaction, as well as the factors that influence them.	Mixed-methods: survey, focus group discussion and in-depth interviews	Overall, 48.6% of HEWs were satisfied with their job. HEWs in the age category of 30 years and older had lower satisfaction scores compared to 18–24 years. Desire to help their community, recognition or respect from the community, and achievement were the major motivating factors. In contrast, inadequate pay and benefit, limited education and career advancement

						opportunities, workload, work environment, limited supportive supervision, and absence of opportunity to change workplace were the demotivating factors.
17	Woldemichael et al, 2019 [17]	Ethiopia	Availability and inequality in accessibility of health centre-based primary healthcare in Ethiopia	Assess availability and measure magnitude and trend of inequalities in accessibility of health centre-based PHC resources in Ethiopia during 2015 to 2017.	Cross-sectional	Availability-The median Skilled Health Workers(SHWs) per health centre was lowest for health officers in Afar and highest for nurses in Dire-Dawa. The median overall SHWs per 10,000 inhabitants in Afar, Tigray, and Dire-Dawa were 5.250 , 6.246 , and 7.539 respectively. Accessibility- Tigray and Dire-Dawa had lower Gini values for all the distributions than the values for the Afar.
18	Avan et al, 2021 [18]	Ethiopia	Embedding Community-Based Newborn Care in the Ethiopian health system: lessons from a 4-year programme evaluation	Evaluate the health system response to the programme, including quality of care.	Before-and-after survey at three timepoints	Results showed gains in services for young infants, with antibiotics and job aids available at over 90% of health centres. Only 37% of CHWs correctly diagnosed key conditions in sick young infants at midline. CHWs' functional health literacy declined by over 70% in basic aspects of case management during the study. Infrastructure and resources improved over the course of the CBNC programme implementation.
19	Argaw et al, 2021 [19]	Ethiopia	Implementing a social accountability approach for maternal, neonatal, and child health service performances in Ethiopia: A pre-post study design	Assess the effects of community score cards implemented by primary health care units (PHCUs) on health system performance in Ethiopia.	Pre-post survey	The use of CSCs in Ethiopia contributed to the health system's performance in terms of maternal and child health services. Of the 10 key maternal neonatal and child health performance indicators measured, 9 were found to improve as a result of implementing the community scorecard approach. The responsiveness of health workers and utilization of basic health services by community members were found to increase significantly with CSC use.
20	Atnafu et al, 2020 [20]	Ethiopia	Community-based health insurance enrollment and child health service utilization in	Examine the association between CBHI enrollment and child health service utilization in northwest Ethiopia.	Cross-sectional case comparison	The overall sick child healthcare visit in the CBHI enrolled group was about 0.44 (44%) point more compared to those unenrolled households. CBHI enrolled households in the poorest wealth group have a higher probability of visiting healthcare facilities for

21			Northwest Ethiopia: A cross-sectional case comparison study			their sick children, whereas CBHI enrolled households with older age household head have a lower probability of visiting healthcare facilities for their sick children.
21	Kebede et al, 2020 [21]	Ethiopia	Evaluation of quality and use of health management information system in primary health care units of east Wollega zone, Oromia regional state, Ethiopia	Evaluate the quality of HMIS data (content Completeness, accuracy, and timeliness) at primary health care units (PHCUs) in East Wollega Zone, Oromia regional state, Ethiopia.	Mixed-methods: cross-sectional study and qualitative focus group discussions	Commonly reported reasons for the poor practice of data quality were; poor support of management, lack of accountability for the false report, poor supportive supervision, and lack of separate and responsible unit for health information management.
22	Desta et al, 2020 [22]	Ethiopia	Leadership, governance and management for improving district capacity and performance: the case of USAID transform: primary health care	Compare capacities and performances between Leadership, management and governance (LMG) and non-LMG districts	Comparative-cross sectional	LMG capacity building addresses the gaps that exist in managers and health workforces at the primary health care level to facilitate equity and quality health services. District level leadership development program contributes to improving district capacity, structure and management practices, and quality of care. LMG districts scored better average performances of compared to non-LMG districts.
23	Desta et al, 2017 [23]	Ethiopia	Identifying gaps in the practices of rural health extension workers in Ethiopia: A task analysis study	Gather information about the frequency, criticality, training, and performance of tasks by rural HEWs in order to identify gaps and recommend changes in their pre-service education, in-service training and scope of practice.	Cross-sectional	A total of 82 rural HEWs participated in the study. HEWs were insufficiently prepared during pre-service education for all tasks that fall within their scope of practice. Many learned tasks through in-service or on-the-job training, and some tasks were not learned at all. Some tasks that are part of expected HEW practice were performed infrequently, potentially reducing the effectiveness of the Health Extension Program to provide preventive and basic curative health care services to communities. Nearly all HEWs rated every

						task as highly critical to individual and public health outcomes.
24	Medhanyie et al, 2017 [24]	Ethiopia	Quality of routine health data collected by health workers using smartphone at primary health care in Ethiopia	Compare the completeness and accuracy of patient data collected using electronic forms on smartphones to that collected by paper forms, by 25 health workers over a period of six months in the Tigray region of Ethiopia.	Comparative cross sectional	With minimal training, supervision, and no incentives, health care workers were able to use electronic forms for patient assessment and routine data collection appropriately and accurately with a very small error rate, electronic forms data completeness 99.2% vs 91.3% paper record entries were complete.
25	Medhanyie et al, 2015 [25]	Ethiopia	Health workers' experiences, barriers, preferences and motivating factors in using mHealth forms in Ethiopia	Assess health workers' experiences, barriers, preferences and motivating factors in using mobile health forms on smartphones in the context of maternal health care in Ethiopia.	Qualitative	Both health extension workers and midwives found the electronic forms on smartphones useful for their day-to-day maternal health care services delivery. Certain barriers to electronic forms were related to the application, smartphone and length of form, requiring technical support, internet, workload etc.
26	Lama et al, 2020 [26]	Nepal	Assessment of facility and health worker readiness to provide quality antenatal, intrapartum and postpartum care in rural Southern Nepal	Assess the facility readiness and health worker knowledge on maternal and newborn care	Cross-sectional	Adequate care was sometimes further delayed due to shortages of drugs and supplies resulting in referral or the need for the family or patient to purchase the supplies and medicines.

27	Gurung et al, 2019 [27]	Nepal	The role of social audit as a social accountability mechanism for strengthening governance and service delivery in the primary health care setting of Nepal: a qualitative study	Explore the role of social audit in facilitating direct accountability between service providers and community in Dang District, Nepal.	Qualitative	Social audit process was able to facilitate information provision/data collection, and provided opportunities for dialogue between community and service providers, had a positive role in increasing transparency, accessibility and quality of services. The provision of sanctions was found to be weak and its effectiveness in addressing perennial governance problems was mixed. Manipulation of the participation process, falsification of information, and lack of authority affected the role of social audit in facilitating accountability.
28	Gurung et al, 2018 [28]	Nepal	Citizen's Charter in a primary health-care setting of Nepal: An accountability tool or a "mere wall poster"?	To gauge the level of awareness of the Charter within Nepal's primary health- care (PHC) system, perceived impact and factors affecting Charter implementation.	Mixed-methods: survey and qualitative interviews	Few service users (15%) were aware of the existence of the Charter. The Charter was usually not properly displayed and had been implemented with no prior public consultation. It contained information that provided awareness of health facility services, particularly the more educated public, but had limited potential for increasing transparency and holding service providers accountable to citizens.
29	Panday et al, 2017 [29]	Nepal	The contribution of female community health volunteers (FCHVs) to maternity care in Nepal: A qualitative study	Explore the role of FCHVs in maternal healthcare provision in two regions: the Hill and Terai in Nepal	Qualitative	All study participants acknowledged the contribution of FCHVs in maternity care. The role of FCHVs varies according to the context in which they work. FCHVs in the hill villages reported activities such as assisting with childbirth, distributing medicines and administering pregnancy tests. They also reported use of innovative approaches to educate mothers. Such activities were not reported in Terai. All FCHVs reported that they shared key health messages through regularly held mothers' group meetings and referred women for health checks.
30	Thapa et al, 2016 [30]	Nepal	Access to Drugs and Out of Pocket Expenditure in	Investigate access to medicines and health care services and Out of Pocket (OOP) expenditure	Cross sectional	Around 91 % of medicines prescribed were dispensed. Out of total medicines dispensed, 86.5% of medicines were found to be adequately labeled and around 84% of service recipients had proper knowledge of medicine

			Primary Health Facilities	endured by outpatients receiving health care from public primary facilities in the post Free Health Care Program(FHCP) context		use. Out of the total, 33.3% of respondents reported medicines shortage while more than half of the outpatients visiting these facilities faced OOP
31	Sato et al, 2015 [31]	Nepal	Exploring health facilities' experiences in implementing the free health-care policy (FHCP) in Nepal: how did organizational factors influence the implementation of the user-fee abolition policy?	Present an Asian experience of abolishing health-care user fees: Nepal's universal free health-care policy, implemented in 2008.	Mixed-methods: document reviews, key informant interviews, in-depth, semi-structured interviews and group interviews, direct observation and field notes	The study identified community governance and support groups such as Health Management Committees (HMCs) as important actors outside the facilities that exercise agency and that could support or hinder the implementation of centrally driven reform.
32	Karki et al, 2015 [32]	Nepal	Health system actors' participation in primary health care in Nepal	Understand how various Health System Actors participate in PHC in Nepal and what its implications are in PHC	Qualitative	There was very low understanding about PHC and community partcipation (CP) among actors in these VDCs. Often, CP for these actors was a 'tokenistic participation' which was limited to material contribution, voluntary labour and financial donation in PHC infrastructure development and maintenance. Participation in Health Facility Management Committees and Female Community Health Volunteer were the only mechanisms of CP in PHC, which rarely represented community views. The main motivations for CP amongst participants were material benefit, social recognition and religious merits whereas geography, opportunity cost, lack of awareness and

						socio-cultural discrimination, were barriers to participation.
33	Baral et al, 2013 [33]	Nepal	Distribution and skill mix of health workforce in Nepal	Generate evidences on the current distribution and skill mix of health workforce in Nepal.	Mixed-methods: cross-sectional and qualitative studies	Proper skill mix was observed in 6 (43%) of hospitals and 3(18%) of PHCCs. The vacant positions are mostly of doctors 74(38%) technicians 28 (21%) nurses 50 (10%) and paramedics 26 (6%) with respect to sanctioned positions. Variations of fulfilled positions occurred in all three ecological belts, with the Hill belt having the highest proportion of vacant posts 116 (16%).On the basis of types of health facilities, ayurvedic centres have the highest fulfilled positions 55 (95%) and the lowest in primary healthcare centres (PHCCs) 162 (81%).
34	Naidoo et al, 2023 [34]	South Africa	New Medicine Service by Community Pharmacists: An Opportunity to Enhance Universal Health Coverage at a Primary Health Level in South Africa	Explore the provision of new medicine service (NMS) within the UHC primary healthcare service package and the opportunity for enhancing pharmacist practice. This pilot reports on the implementation of NMS in a low-middle income country.	Pilot quantitative study	Out of the 35 patients (n = 35) that completed the program, 65.71% (n = 23) had no problems detected by the end; rather the program served as a platform to provide information and ensure proper initiation of new chronic medication. Twelve (34.29%) patients experienced problems and were referred back to the prescriber, or pharmacist intervention was required after follow-up visits. After the completion of the program by all patients, 54.29% (n = 19) where found to be adequately adherent to the newly prescribed medication based on evaluation of their medicine-taking practices, structured interviews, and questionnaire; and exited the program. However, 45.71% (n = 16) were found to be non-adherent after completion of the program and were counseled accordingly or referred back to the medical practitioner.
35	Haripersad & Bangalee, 2022 [35]	South Africa	Availability of medical schemes' formulary medicines in community	Determine how many formulary medicines from a basket of 24 active ingredients are available for sale in different community	Cross-sectional	At least one medicine brand per active ingredient was found to be available at each pharmacy, representing 100% availability of all surveyed active ingredients for this study.

			pharmacies in Durban, South Africa	pharmacies in the eThekwini Metropolitan Municipality of KwaZuluNatal		
36	Malatji & Goudge, 2023 [36]	South Africa	Community-orientated primary health care: exploring the interface between community health worker programmes, the health system and communities in South Africa	Explore to what extent a nation-wide CHW 21 programme in South Africa is attuned to community needs, integrated into the healthcare system and 22 community structures, and also implemented in accordance with community-orientated primary 23 health care principles.	Qualitative	There was some evidence of resistance by community members to participate in the design and implementation of the health programmes, as the residents were more interested in housing than healthcare. Also, CHWs insufficient health knowledge and skills contributed to the ineffectiveness of the programmes, particularly were the CHWs did not have access to a senior team leader to provide 441 them with supervision and mentorship. However, the multidisciplinary approach to care which saw 442 medical doctors providing in-service training to CHWs in some sites, improving the services provided to patients. As much as there was an attempt to implement the programmes in accordance with COPC principles, dysfunctional mobile phones for patient data collection, volatile communities and an unsupportive healthcare system limited the effectiveness of the programmes to deliver care.
37	Mash et al, 2022 [37]	South Africa	Retention of medical officers in district health services, South Africa: a descriptive survey	To evaluate factors that influence retention of medical officers in public sector DHS.	Cross-sectional survey	The overall rating of the facility was one of the key factors in determining retention. Factors related to social and family life were important. Those who planned to specialise, emigrate, enter the private sector, or change professions were also more likely to leave. This study suggested that teamwork, support, and supervision were critical.

38	Murphy et al, 2021 [38]	South Africa	Community health worker models in South Africa: a qualitative study on policy implementation of the 2018/19 revised framework	Document the CHW policy implementation landscape across six provinces in South Africa and explore the reasons for local adaptation of CHW models and to identify potential barriers and facilitators to implementation of the revised framework to help guide and inform future planning.	Qualitative	Strong consistent messages on health priorities, supervision and guidance, sense of purpose, trust from the community, supportive relationship with facility management were seen as key facilitators for CHWs. Barriers were related to allocation of CHWs to wards, wage, professional development opportunities, limited resources and workload.
39	Haricharan et al, 2021 [39]	South Africa	The role of community participation in primary health care: practices of South African health committees	Analyse health committees' roles, their degree of influence in decision-making and factors impacting their participation.	Mixed-methods: cross-sectional survey, focus groups, interviews and observations.	The study found that 55 per cent ofclinics in Cape Town were linked to a health committee. The existing health committees faced sustainability and functionality challenges and primarily practised a form oflimited participation. Their decision-making influence was curtailed, and they mainly functioned as a voluntary workforce assisting clinics with health promotion talks and day-to-day operational tasks. Several factors impacted health committee participation, including lack of clarity on health committees' roles, health committee members' skills, attitudes offacility managers and ward councillors, limited resources and support and lack ofrecognition.
40	Jobson et al, 2020 [40]	South Africa	Contextual factors affecting the integration of community health workers into the health system in Limpopo Province, South Africa	Assess contextual factors that shape the implementation of the WBOT programme in the Mopani district of Limpopo province in order to contribute to a more in-depth understanding ofthe	Qualitative	Six critical contexts affecting the implementation of the WBOT programme were identified: geographic, social and economic, community, local governance and authority, and organisational. CHWs reported that where community leaders were supportive and engaged with them, this support facilitated easier access to households in their communities. Several critical factors related to the organisational context of the CHW

				ways in which CHWs interact with and integrate into the formal health system.		programme, these included tensions between the NDoH and non-governmental organisations (NGOs) responsible for paying CHWs, staff shortages within the NDoH and the lack of formally designated WBOT leader posts, the overburdening of CHWs in terms of their reporting requirements and the lack of effective integration of social workers into the WBOTs' operational strategies.
41	Majee et al, 2020 [41]	South Africa	Emerging from the shadows: Intrinsic and extrinsic factors facing community health workers in Western Cape, South Africa	Explore intrinsic and extrinsic factors that CHWs face.	Qualitative	Based on data, two main challenge themes (a) intrinsic factors (e.g., CHWs coping with their own emotional, physical, and psychological challenges and gender sensitivity challenges) and (b) extrinsic factors (e.g., lack of understanding of the CHW role by various stakeholders; environmental challenges: lack of resources in terms of medical supplies, lack of transport; lack of patient and family adherence) emerged
42	Ndaba et al, 2020 [42]	South Africa	Establishing a community advisory group (CAG) for partnership defined quality (PDQ) towards improving primary health care in a peri-urban setting in KwaZulu-Natal, South Africa	Explore the establishment, operation, and accomplishments of a community advisory grouo (CAG) towards building a strong partnership between the health facilities and local communities, to improve the delivery of quality maternal and neonatal care	Qualitative	The CAG used four indicators (weaknesses, threats and risks, strengths, and opportunities) to review the community and primary health care challenges that affect their communities. The CAG aimed o create an enabling environment for all stakeholders, especially the teams working on improving the care of pregnant mothers and their babies.
43	Iwu et al, 2020 [43]	South Africa	Mobile reporting of vaccine stock-levels in primary health care facilities in the Eastern Cape Province of South	Aimed to explore the perceptions and experiences of the Stock Visibility Solution (SVS) system amongst healthcare workers (HCWs) who are	Qualitative	While HCWs were committed to addressing vaccine stock-outs, they faced various barriers to an effective and efficient implementation of the SVS system: inadequate training, staff shortages and high staff turnover, lack of responses from the managers, the extra workload that comes with the system, amongst others.

			Africa: perceptions and experiences of health care workers	involved with managing stock levels of medicines in primary health care facilities in the Eastern Cape Province.		HCWs suggested the need for more pharmacists and pharmacy assistants, and them to be primarily in-charge of stock management and the use of the SVS.
44	GSM Association, 2018 [44]	South Africa	Mezzanine's Stock Visibility Solution A mobile solution driving increased access to medicines	Describe broader strategy and portfolio of digital health tools, and demonstrates how the company has leveraged public-private partnerships (PPPs) to provide holistic digital health solutions at national scale	Report	Mezzanine's Stock Visibility Solution (SVS) enables health facilities to transition from inefficient paper-based stock reporting processes to digital data collection and real-time reporting. SVS reduces stockouts and expands public access to essential medicines and vaccines.
45	Ndzamela, 2020 [45]	South Africa	Patients and healthcare professionals' experiences of medicine stock-outs and shortages at a community healthcare centre in the Eastern Cape	Explore and describe both patients and healthcare professionals' experiences of medicine stockouts and shortages at a primary healthcare centre in the Nelson Mandela Metropole.	Qualitative	Due to stock-outs and shortages:<br>Patient experiences included dissatisfaction, anger, frustration and a loss of confidence in the healthcare service.<br>Medicine stock-outs resulted in stress and burn out in healthcare professionals.<br>Healthcare professionals also experienced violence and verbal abuse from patients because of medicine stock-outs.<br>Inadequate administration and lack of communication were perceived as significant causes of stock-outs, which negatively influenced relationships between healthcare professionals and patients.
46	Michel et al, 2020 [46]	South Africa	What we need is health system transformation and not health system strengthening for universal health	Understand how policy–practice gaps come about in a UHC context.	Qualitative	Lack of resources to transform policy into action was a major stumbling block. The key informants shared the top-down nature of NHI policies affected their motivation, as some policies were found not relevant by some facilities, and necessary equipment like vaccine fridges were not supplied. Many actors cited being

			coverage to work: Perspectives from a National Health Insurance pilot site in South Africa			summoned to the district for meetings, taking them away from work and delaying policy implementation. The lack of power to take decisive actions, for example, to hire personnel or get resources, was cited as a huge stumbling block in policy implementation.
47	Bresick & Mash, 2019 [47]	South Africa	Evaluating the performance of South African primary care: a cross-sectional descriptive survey	Evaluate the quality of South African primary care using the Primary Care Assessment Tool (PCAT).	Cross-sectional survey	Patients rated first contact accessibility, ongoing care and community orientation as the poorest performing elements (< 50% scoring as 'acceptable to good'); first contact utilisation, informational coordination and family-centredness as weaker elements (< 66% scoring as 'acceptable to good'); and comprehensiveness, coordination, cultural competency and availability of the PHC team as stronger aspects of primary care (≥ 66% or more scoring as 'acceptable or good'). Managers and providers were generally much more positive about the performance of PHC.
48	Girdwood et al, 2019 [48]	South Africa	Primary healthcare delivery models for uninsured low-income earners during the transition to National Health Insurance: Perspectives of private South African providers	Describe the current private PHC delivery landscape outside the traditional GP model of private PHC.	Qualitative	Of the eight organisations identified, most actively engaged strategies to ensure the provision of affordable quality care such as, spreading fixed costs across more paying patients as well as task shifting to lower cadres of healthcare workers, access to government medicines and laboratory tests to achieve lower costs per patient.
49	Meyer et al, 2018 [49]	South Africa	Why high tech needs high touch: Supporting continuity of	Understand how household to clinical facility-integrated community oriented primary care (COPC), using	Mixed methods: quantitative and qualitative data collection	An integrated COPC approach increased the number of traceable pregnant women followed up at home from 2016 – 2017. Wrong addresses or personal identification were given at the clinic because of fear of being denied care. Allocating patients correctly to a ward-based

			community primary health care	ICT and working with CHWs, can effectively be combined with routine clinical antenatal care in improving antenatal, postnatal, and maternal and child care.		outreach team (WBOT) proved to be a challenge as many patients did not know their street address.
50	Assegaai et al, 2017 [50]	South Africa	Evaluating the effect of ward-based outreach teams on primary healthcare performance in North West Province, South Africa: A plausibility design using routine data	Describe the application of a plausibility evaluation design for assessing the contribution of WBOTs to PHC performance	Secondary data analysis	WBOTs plausibly had some positive effects on the overall performance of the PHC system. Changes in indicator values in facilities were grouped into four categories: (i) indicators where there was greater (statistically significant) improvement in facilities with WBOTs (couple year protection rate, measles immunisation coverage in children aged <1 year, incidence of children aged <5 years with severe diarrhoea with dehydration); (ii) indicators that declined or worsened, but less so in facilities with WBOTs at statistically significant levels (antenatal first visits as a percentage of children born in that year, PHC utilisation rate of children aged <5 years); (iii) indicators that improved in all facilities with no significant difference between facilities with and without WBOTs (antenatal attendance before 20 weeks, prophylactic vitamin A coverage to children aged 12 - 59 months); and (iv) indicators that remained unchanged in all facilities (immunisation coverage in children aged <1 year, postnatal mother visits at 6 days, cervical cancer screening coverage in women aged ≥30 years, PHC utilisation rate of children aged ≥5 years).
51	Moosa S & Gibbs A 2014 [51]	South Africa	A focus group study on primary health care in Johannesburg	Explore Johannesburg stakeholder views on clinical priorities, the role	Qualitative	Addressing staff burnout and poor management were viewed as clinical priorities in primary care. Discussing the role of doctors reflected deep conflict between doctors and nurses. Nurses and managers expected

			Health District: We are just pushing numbers	of doctors and family medicine in primary care		doctors to help to "push the queues". It took some time for further roles, such as helping with referrals, training, research and administration, to emerge. There was initial confusion and tension when participants were asked about family medicine. However, its role was seen as useful.
52	Visagie, S. & Schneider, M., 2014 [52]	South Africa	Implementation of the principles of primary health care in a rural area of South Africa	Explore the extent to which the principles of PHC are implemented in a remote, rural setting in South Africa, as well as to identify gaps and possible solutions to the implementation of PHC in this setting.	Qualitative	Findings indicated challenges with regard to client-centred care, provision of health promotion and rehabilitation, the way care was organised, the role of the doctor, healthworker attitudes, referral services and the management of complex conditions. The principles of primary healthcare were not implemented successfully. The community was not involved in healthcare management, nor were users involved in their personal health management. The initiation of a community-health forum is recommended. Service providers, users and the community should identify and address the determinants of ill health in the community. Other recommendations include the training of service managers in the logistical management of ensuring a constant supply of drugs, using a Kombi-type vehicle to provide user transport for routine visits to secondary- and tertiary healthcare services and increasing the doctors' hours.
53	Schneider et al, 2014 [53]	South Africa	Whole-system change: case study of factors facilitating early implementation of a primary health care reform in a South African province	Present the contextual factors and implementation strategies that enabled the uptake of the PHC outreach team strategy in the North West Province	Qualitative	Implementation of the PHC outreach team strategy was characterised by the following features: 1) A favourable provincial context of a well established district and sub-district health system and long standing values in support of PHC; 2) The forging of a collective vision for the new strategy that built on prior history and values and that led to distributed leadership and ownership of the new policy; 3) An implementation strategy that ensured alignment of systems (information, human

						resources) and appropriate sequencing of activities (planning, training, piloting, household campaigns); 4) The privileging of 'community dialogues' and local manager participation in the early phases; 5) The establishment of special implementation structures: a PHC Task Team (chaired by a senior provincial manager) to enable feedback and ensure accountability, and an NGO partnership that provided flexible support for implementation.
54	Mckenzie & Mccann, 2016 [54]	South Africa	Evaluation of the Stop Stock outs Project (SSP). South Africa: Médecins sans Frontières (MSF); 2016	To evaluate the impact on provincial/national government policy and reform, on community mobilisation to address stock outs, on creating an effective advocacy consortium on health and HIV, on strengthening a regional stop stock outs movement and on assessing replicability regionally and in other service areas	Mixed methods: document review, interviews, group discussions, observations	A combination of a community mobilisation strategy, communication initiatives, and a hotline for health care workers and patients to report drug stockouts successfully ad-dressed stockout locally through community and health care workers' collaboration. Other factors contributing to stockouts were inadequate administration and lack of communication at PHC centres.
55	Kitreerawutiwong et al, 2018 [55]	Thailand	Assessing the implementation of the family care team in the district health system of health region 2, Thailand	This study aimed to explore the implementation of the family care team (FCT) policy and describe the challenges in implementing the FCT in the DHS	Qualitative	The vulnerable group gained access to health care service more easily and received more continuous care. This is due to the FCT working through family medicine principles. There were considerable changes in the FCT in the provision of care because the local organization and local people were invited to participate as team members working with the health sector. Internal communication and communication among FCTs became problematic for collaboration. The

						team members were confused about the manager's role because when the FCT was implemented, the focus was on the role of each professional in the FCT.
56	Suphanchaimat et al, 2016 [56]	Thailand	Health insurance for people with citizenship problems in Thailand: A case study of policy implementation	This study focused on the operational constraints faced by ground level providers in policy implementation of health insurance scheme for people with citizenship problems	Qualitative	Inadequate communication and unclear service guidelines contributed to ineffectiveness in budget spend and service provision. Other problems included the legal instruments that permitted stateless people to live only in certain areas, when such people were in fact highly mobile. Some providers adapted their practices to cope with on-the-job difficulties, including establishing a mutual agreement with neighbouring hospitals to allow stateless patients to bypass primary care gatekeepers. The challenges were aggravated by the delays in nationality verification procedures and insufficient collaboration between the Ministry of Public Health (MOPH) and the Ministry of Interior.
57	Sirilak et al, 2013 [57]	Thailand	Community participation of cross-border migrants for primary health care in Thailand	Explore migrant volunteers, their relationship with the management structure of the program and their attitudes towards volunteer work.	Cross-sectional	Recruitment of migrants into the primary healthcare system can be beneficial. Attitudes towards the volunteer program were very positive, and the migrants understood the benefits of their volunteers and utilized health services. A quarter of them (25.4%) reported problems with voluntary work; the reasons included lack of knowledge (33.3%), insufficient time (34.8%), communication problems (24.2%) and too much responsibility (10.6%).
58	Yiengprugsawan et al, 2010 [58]	Thailand	Has universal health insurance reduced socioeconomic inequalities in urban and rural health service use in Thailand?	Describe the patterns of urban and rural use of health services before and after the Universal Coverage Scheme (UCS); second, to quantify and assess the magnitudes of inequalities in health	Secondary data analysis	Substantial declines in proportions of respondents with no health insurance coverage occurred between 2001 and 2005 in both urban and rural areas. After the introduction of the UCS, 23.6% of rural respondents in 2005 were covered by the UCS with fee exempt, compared to only 8.9% of urban respondents. Pharmacy use was disproportionately concentrated among the better off in both urban and rural areas at both dates,

				service use before and after the UCS; third, to compare the patterns and inequalities in health service use between urban and rural areas before and after the UCS.		but the measured socioeconomic inequalities were all small.

References

1. Mathew, S.; Fitts, M. S.; Liddle, Z.; Bourke, L.; Campbell, N.; Murakami-Gold, L.; Russell, D. J.; Humphreys, J. S.; Mullholand, E.; Zhao, Y.; et al. Telehealth in remote Australia: a supplementary tool or an alternative model of care replacing face-to-face consultations? *BMC Health Serv Res* 2023, *23* (1), 341. https://doi.org/10.1186/s12913-023-09265-2
2. Ashley, C.; Williams, A.; Dennis, S.; McInnes, S.; Zwar, N. A.; Morgan, M.; Halcomb, E. Telehealth's future in Australian primary health care: a qualitative study exploring lessons learnt from the COVID-19 pandemic. *BJGP Open* 2023, *7* (2). https://doi.org/10.3399/BJGPO.2022.0117
3. Nolan-Isles, D.; Macniven, R.; Hunter, K.; Gwynn, J.; Lincoln, M.; Moir, R.; Dimitropoulos, Y.; Taylor, D.; Agius, T.; Finlayson, H.; et al. Enablers and Barriers to Accessing Healthcare Services for Aboriginal People in New South Wales, Australia. *Int J Environ Res Public Health* 2021, *18* (6), 1-13. https://doi.org/10.3390/ijerph18063014
4. Butler, D. C.; Jorm, L. R.; Larkins, S.; Humphreys, J.; Desborough, J.; Korda, K. J. Examining area-level variation in service organisation and delivery across the breadth of primary healthcare. Usefulness of measures constructed from routine data. *PLoS One* 2021, *16* (12), e0260615. https://doi.org/10.1371/journal.pone.0260615
5. Topp, S. M.; Tully, J.; Cummins, R.; Graham, V.; Yashadhana, A.; Elliott, L.; Taylor, S. Unique knowledge, unique skills, unique role: Aboriginal and Torres Strait Islander Health Workers in Queensland, Australia. *BMJ Glob Health* 2021, *6* (7). https://doi.org/10.1136/bmjgh-2021-006028
6. Freeman, T.; Baum, F.; Lawless, A.; Javanparast, S.; Jolley, G.; Labonte, R.; Bentley, M.; Boffa, J.; Sanders, D. Revisiting the ability of Australian primary healthcare services to respond to health inequity. *Aust J Prim Health* 2016, *22* (4), 332-338. https://doi.org/10.1071/PY14180
7. Reeve, C.; Humphreys, J.; Wakerman, J.; Carroll, V.; Carter, M.; O'Brien, T.; Erlank, C.; Mansour, R.; Smith, B. Community participation in health service reform: the development of an innovative remote Aboriginal primary health-care service. *Aust J Prim Health* 2015, *21* (4), 409-416. https://doi.org/10.1071/PY14073
8. Anaf, J.; Baum, F.; Freeman, T.; Labonte, R.; Javanparast, S.; Jolley, G.; Lawless, A.; Bentley, M. Factors shaping intersectoral action in primary health care services. *Aust N Z J Public Health* 2014, *38* (6), 553-559. https://doi.org/10.1111/1753-6405.12284
9. Russell, D. J.; McGrail, M. R.; Humphreys, J. S.; Wakerman, J. What factors contribute most to the retention of general practitioners in rural and remote areas? *Aust J Prim Health* 2012, *18* (4), 289-294. https://doi.org/10.1071/PY11049
10. Javanparast, S.; Baum, F.; Freeman, T.; Ziersch, A.; Henderson, J.; Mackean, T. Collaborative population health planning between Australian primary health care organisations and local government: lost opportunity. *Australian and New Zealand Journal of Public Health* 2019, *43* (1), 68-74. https://doi.org/10.1111/1753-6405.12834
11. Masquillier, C.; Cosaert, T. Facilitating access to primary care for people living in socio-economically vulnerable circumstances in Belgium through community health workers: towards a conceptual model. *BMC Primary Care* 2023, *24* (1), 281. https://doi.org/10.1186/s12875-023-02214-2
12. Asante, A. D.; Ir, P.; Jacobs, B.; Supon, L.; Liverani, M.; Hayen, A.; Jan, S.; Wiseman, V. Who benefits from healthcare spending in Cambodia? Evidence for a universal health coverage policy. *Health Policy Plan* 2019, *34* (Supplement_1), i4-i13. https://doi.org/10.1093/heapol/czz011
13. Chhea, C.; Warren, N.; Manderson, L. Health worker effectiveness and retention in rural Cambodia. *Rural Remote Health* 2010, *10* (3), 1391. (accessed 15 January 2024).

14. Yitbarek, K.; Serawit, A.; Medhin, G.; Alemayehu, Y. K.; Teklu, A. M.; Assefa, Y.; Haregu, T. N. Capacity of the Ethiopian primary health care system to achieve universal health coverage: a primary health care progression approach. *Health Policy Plan* 2023, *38* (4), 474-485. https://doi.org/10.1093/heapol/czad013
15. Hailemariam, T.; Atnafu, A.; Gezie, L. D.; Kaasboll, J. J.; Klein, J.; Tilahun, B. Individual and contextual level enablers and barriers determining electronic community health information system implementation in northwest Ethiopia. *BMC Health Serv Res* 2023, *23* (1), 644. https://doi.org/10.1186/s12913-023-09629-8
16. Ejigu, Y.; Abera, N.; Haileselassie, W.; Berhanu, N.; Haile, B. T.; Nigatu, F.; Tewfik, N.; Kiflie, Y.; Medhin, G.; Walelign, F.; et al. Motivation and job satisfaction of community health workers in Ethiopia: a mixed-methods approach. *Hum Resour Health* 2023, *21* (1), 35. https://doi.org/10.1186/s12960-023-00818-4
17. Woldemichael, A.; Takian, A.; Akbari Sari, A.; Olyaeemanesh, A. Availability and inequality in accessibility of health centre-based primary healthcare in Ethiopia. *PLoS One* 2019, *14* (3), e0213896. https://doi.org/10.1371/journal.pone.0213896
18. Avan, B. I.; Berhanu, D.; Mekonnen, Y.; Beaumont, E.; Tomlin, K.; Allen, E.; Schellenberg, J. Embedding Community-Based Newborn Care in the Ethiopian health system: lessons from a 4-year programme evaluation. *Health Policy Plan* 2021, *36* (Supplement_1), i22-i32. https://doi.org/10.1093/heapol/czab085
19. Argaw, M. D.; Fekadu, B. D.; Mamo, E.; Abebe, M. G.; Rogers, D.; Demelash, A.; Ayele, A. A.; Reda, Z.; Tareke, A. S.; Erfo, A. M.; et al. Implementing a Social Accountability Approach for Maternal, Neonatal, and Child Health Service Performances in Ethiopia: A Pre-Post Study Design. *Glob Health Sci Pract* 2021, *9* (1), 123-135. https://doi.org/10.9745/GHSP-D-20-00114
20. Atnafu, A.; Gebremedhin, T. Community-Based Health Insurance Enrollment and Child Health Service Utilization in Northwest Ethiopia: A Cross-Sectional Case Comparison Study. *Clinicoecon Outcomes Res* 2020, *12*, 435-444. https://doi.org/10.2147/CEOR.S262225
21. Kebede, M.; Adeba, E.; Chego, M. Evaluation of quality and use of health management information system in primary health care units of east Wollega zone, Oromia regional state, Ethiopia. *BMC Medical Informatics and Decision Making* 2020, *20* (1), 107. https://doi.org/10.1186/s12911-020-01148-4
22. Desta, B. F.; Abitew, A.; Beshir, I. A.; Argaw, M. D.; Abdlkader, S. Leadership, governance and management for improving district capacity and performance: the case of USAID transform: primary health care. *BMC Fam Pract* 2020, *21* (1), 252. https://doi.org/10.1186/s12875-020-01337-0
23. Desta, F. A.; Shifa, G. T.; Dagoye, D. W.; Carr, C.; Van Roosmalen, J.; Stekelenburg, J.; Nedi, A. B.; Kols, A.; Kim, Y. M. Identifying gaps in the practices of rural health extension workers in Ethiopia: a task analysis study. *BMC Health Serv Res* 2017, *17* (1), 839. https://doi.org/10.1186/s12913-017-2804-0
24. Medhanyie, A. A.; Spigt, M.; Yebyo, H.; Little, A.; Tadesse, K.; Dinant, G. J.; Blanco, R. Quality of routine health data collected by health workers using smartphone at primary health care in Ethiopia. *Int J Med Inform* 2017, *101*, 9-14. https://doi.org/10.1016/j.ijmedinf.2017.01.016
25. Medhanyie, A. A.; Little, A.; Yebyo, H.; Spigt, M.; Tadesse, K.; Blanco, R.; Dinant, G. J. Health workers' experiences, barriers, preferences and motivating factors in using mHealth forms in Ethiopia. *Hum Resour Health* 2015, *13* (1), 2. https://doi.org/10.1186/1478-4491-13-2
26. Lama, T. P.; Munos, M. K.; Katz, J.; Khatry, S. K.; LeClerq, S. C.; Mullany, L. C. Assessment of facility and health worker readiness to provide quality antenatal, intrapartum and postpartum care in rural Southern Nepal. *BMC Health Serv Res* 2020, *20* (1), 16. https://doi.org/10.1186/s12913-019-4871-x
27. Gurung, G.; Derrett, S.; Hill, P. C.; Gauld, R. The role of social audit as a social accountability mechanism for strengthening governance and service delivery in the primary health care setting of Nepal: a qualitative study. *Critical Public Health* 2019, *30* (5), 612-623. https://doi.org/10.1080/09581596.2019.1667487

28. Gurung, G.; Gauld, R.; Hill, P. C.; Derrett, S. Citizen's Charter in a primary health-care setting of Nepal: An accountability tool or a "mere wall poster"? *Health Expect* 2018, *21* (1), 149-158. https://doi.org/10.1111/hex.12596
29. Panday, S.; Bissell, P.; van Teijlingen, E.; Simkhada, P. The contribution of female community health volunteers (FCHVs) to maternity care in Nepal: a qualitative study. *BMC Health Serv Res* 2017, *17* (1), 623. https://doi.org/10.1186/s12913-017-2567-7
30. Thapa, A. K.; Ghimire, N.; Adhikari, S. R. Access to Drugs and Out of Pocket Expenditure in Primary Health Facilities. *J Nepal Health Res Counc* 2016, *14* (34), 139-142. https://www.ncbi.nlm.nih.gov/pubmed/28327677 (accessed 16 January 2024).
31. Sato, M.-.-G., L. Exploring health facilities' experiences in implementing the free health-care policy (FHCP) in Nepal: How did organizational factors influence the implementation of the user-fee abolition policy? *Health Policy and Planning* 2015, *30* (10), 1272-1288. https://doi.org/10.1093/heapol/czu136
32. Karki, J. K.; Lee, A.; Johnson, M.; Simkhada, P.; Chhetri, M. R.; Jones, G. Health system actors' participation in primary health care in Nepal. *Tropical Medicine & International Health* 2015, *20*, 373-374. https://doi.org/10.1111/tmi.12574
33. Baral, B.; Prajapati, R.; Karki, K. B.; Bhandari, K. Distribution and skill mix of health workforce in Nepal. *J Nepal Health Res Counc* 2013, *11* (24), 126-132. https://pubmed.ncbi.nlm.nih.gov/24362599/ (accessed 16 January 2024).
34. Naidoo, V.; Moodley, R.; Bangalee, V.; Suleman, F. New Medicine Service by Community Pharmacists: An Opportunity to Enhance Universal Health Coverage at a Primary Health Level in South Africa. *Inquiry* 2023, *60*, 469580221146834. https://doi.org/10.1177/00469580221146834
35. Haripersad, B.; Padayachee, N.; Bangalee, V. Availability of medical schemes' formulary medicines in community pharmacies in Durban, South Africa. *Journal of Pharmaceutical Health Services Research* 2022, *13* (2), 95-103. https://doi.org/10.1093/jphsr/rmac013
36. Malatji, H.; Griffiths, F.; Goudge, J. Community-orientated primary health care: exploring the interface between community health worker programmes, the health system and communities in South Africa. *medRxiv* 2022, *3* (2). https://doi.org/10.1101/2022.07.15.22277684
37. Mash, R.; Williams, B.; Stapar, D.; Hendricks, G.; Steyn, H.; Schoevers, J.; Wagner, L.; Abbas, M.; Kapp, P.; Perold, S.; et al. Retention of medical officers in district health services, South Africa: a descriptive survey. *BJGP Open* 2022, *6* (4). https://doi.org/10.3399/BJGPO.2022.0047
38. Murphy, J. P.; Moolla, A.; Kgowedi, S.; Mongwenyana, C.; Mngadi, S.; Ngcobo, N.; Miot, J.; Evans, D.; Pascoe, S. Community health worker models in South Africa: a qualitative study on policy implementation of the 2018/19 revised framework. *Health Policy Plan* 2021, *36* (4), 384-396. https://doi.org/10.1093/heapol/czaa172
39. Haricharan, H. J.; Stuttaford, M.; London, L. The role of community participation in primary health care: practices of South African health committees. *Prim Health Care Res Dev* 2021, *22*, e31. https://doi.org/10.1017/S146342362100027X
40. Jobson, G.; Naidoo, N.; Matlakala, N.; Marincowitz, G.; Railton, J.; McIntyre, J. A.; Struthers, H. E.; Peters, R. P. H. Contextual factors affecting the integration of community health workers into the health system in Limpopo Province, South Africa. *Int Health* 2020, *12* (4), 281-286. https://doi.org/10.1093/inthealth/ihz082
41. Majee, W.; Schopp, L.; Johnson, L.; Anakwe, A.; Rhoda, A.; Frantz, J. Emerging from the Shadows: Intrinsic and Extrinsic Factors Facing Community Health Workers in Western Cape, South Africa. *Int J Environ Res Public Health* 2020, *17* (9). https://doi.org/10.3390/ijerph17093199
42. Ndaba, T.; Taylor, M.; Mabaso, M. Establishing a community advisory group (CAG) for partnership defined quality (PDQ) towards improving primary health care in a peri-urban setting in KwaZulu-Natal, South Africa. *BMC Health Serv Res* 2020, *20* (1), 392. https://doi.org/10.1186/s12913-020-05275-6

43. Iwu, C. J.; Ngcobo, N.; Cooper, S.; Mathebula, L.; Mangqalaza, H.; Magwaca, A.; Chikte, U.; Wiysonge, C. S. Mobile reporting of vaccine stock-levels in primary health care facilities in the Eastern Cape Province of South Africa: perceptions and experiences of health care workers. *Human Vaccines and Immunotherapeutics* 2020, *16* (8), 1911-1917. https://doi.org/10.1080/21645515.2019.1700713
44. GSM Association. *Mezzanine's Stock Visibility Solution: A mobile solution driving increased access to medicines*; GSM Association, London, 2018. https://www.gsma.com/solutions-and-impact/connectivity-for-good/mobile-for-development/wp-content/uploads/2018/07/GSMA_Mezzanine%E2%80%99s-Stock-Visibility-Solution.pdf (accessed 5 February 2024).
45. Ndzamela, S.; Burton, S. Patients and healthcare professionals' experiences of medicine stock-outs and shortages at a community healthcare centre in the Eastern Cape. *Sa Pharmaceutical Journal* 2020, *87* (5), 37I-37M. https://hdl.handle.net/10520/ejc-mp_sapj-v87-n5-a10
46. Michel, J.; Obrist, B.; Barnighausen, T.; Tediosi, F.; McIntryre, D.; Evans, D.; Tanner, M. What we need is health system transformation and not health system strengthening for universal health coverage to work: Perspectives from a National Health Insurance pilot site in South Africa. *S Afr Fam Pract (2004)* 2020, *62* (1), e1-e15. https://doi.org/10.4102/safp.v62i1.5079
47. Bresick, G.; von Pressentin, K. B.; Mash, R. Evaluating the performance of South African primary care: a cross-sectional descriptive survey. *South African Family Practice* 2019, *61* (3), 109-116. https://doi.org/10.1080/20786190.2019.1596666
48. Girdwood, S.; Govender, K.; Long, L.; Miot, J.; Meyer-Rath, G. Primary healthcare delivery models for uninsured low-income earners during the transition to National Health Insurance: Perspectives of private South African providers. *S Afr Med J* 2019, *109* (10), 771-783. https://doi.org/10.7196/SAMJ.2019.v109i10.13930
49. Meyer, E. D.; Hugo, J. F. M.; Marcus, T. S.; Molebatsi, R.; Komana, K. Why high tech needs high touch: Supporting continuity of community primary health care. *Afr J Prim Health Care Fam Med* 2018, *10* (1), e1-e6. https://doi.org/10.4102/phcfm.v10i1.1616
50. Assegaai, T.; Reagon, G.; Schneider, H. Evaluating the effect of ward-based outreach teams on primary healthcare performance in North West Province, South Africa: A plausibility design using routine data. *S Afr Med J* 2018, *108* (4), 329-335. https://doi.org/10.7196/SAMJ.2017.v108i4.12755
51. Moosa, S.; Gibbs, A. A focus group study on primary health care in Johannesburg Health District: "We are just pushing numbers". *South African Family Practice* 2014, *56* (2), 147-152. https://doi.org/10.1080/20786204.2014.10855353
52. Visagie, S.; Schneider, M. Implementation of the principles of primary health care in a rural area of South Africa. *Afr J Prim Health Care Fam Med* 2014, *6* (1), E1-E10. https://doi.org/10.4102/phcfm.v6i1.562
53. Schneider, H.; English, R.; Tabana, H.; Padayachee, T.; Orgill, M. Whole-system change: case study of factors facilitating early implementation of a primary health care reform in a South African province. *BMC Health Serv Res* 2014, *14*, 609. https://doi.org/10.1186/s12913-014-0609-y
54. Mckenzie, A.; Mccann, T. *Evaluation of the stop stock outs project (SSP)*; Stockholm Evaluation Unit, South Africa, 2016. (accessed 5 February 2024).
55. Kitreerawutiwong, N.; Mekrungrengwong, S.; Wongwonsin, A.; Phetphum, C. Assessing the implementation of the family care team in the district health system of health region 2, Thailand. *Family Medicine and Community Health* 2018, *6* (1), 39-50. https://doi.org/10.15212/Fmch.2017.0139
56. Suphanchaimat, R.; Kantamaturapoj, K.; Pudpong, N.; Putthasri, W.; Mills, A. Health insurance for people with citizenship problems in Thailand: a case study of policy implementation. *Health Policy Plan* 2016, *31* (2), 229-238. https://doi.org/10.1093/heapol/czv046
57. Sirilak, S.; Okanurak, K.; Wattanagoon, Y.; Chatchaiyalerk, S.; Tornee, S.; Siri, S. Community participation of cross-border migrants for primary health care in Thailand. *Health Policy Plan* 2013, *28* (6), 658-664. https://doi.org/10.1093/heapol/czs105

58. Yiengprugsawan, V.; Carmichael, G. A.; Lim, L. L.; Seubsman, S. A.; Sleigh, A. C. Has universal health insurance reduced socioeconomic inequalities in urban and rural health service use in Thailand? *Health Place* 2010, *16* (5), 1030-1037. https://doi.org/10.1016/j.healthplace.2010.06.010
